# Microbiological Reduction of Molybdenum to Molybdenum Blue as a Sustainable Remediation Tool for Molybdenum: A Comprehensive Review

**DOI:** 10.3390/ijerph18115731

**Published:** 2021-05-27

**Authors:** Hafeez Muhammad Yakasai, Mohd Fadhil Rahman, Motharasan Manogaran, Nur Adeela Yasid, Mohd Arif Syed, Nor Aripin Shamaan, Mohd Yunus Shukor

**Affiliations:** 1 Department of Biochemistry, Faculty of Biotechnology and Biomolecular Sciences, Universiti Putra Malaysia, Serdang 43400, Malaysia; hmyakasai.bch@buk.edu.ng (H.M.Y.); fadhilep3@gmail.com (M.F.R.); haranz715@yahoo.com (M.M.); adeela@upm.edu.my (N.A.Y.); marifsyed@gmail.com (M.A.S.); 2 Department of Biochemistry, Faculty of Basic Medical Sciences, College of Health Science, Bayero University, Kano PMB 3011, Nigeria; 3 Faculty of Medicine and Health Sciences, Universiti Sains Islam Malaysia, Kuala Lumpur 55100, Malaysia; naripin@usim.edu.my

**Keywords:** molybdenum, bioremediation, molybdenum-reducing bacterium, molybdenum blue, inhibition kinetics, enzyme purification

## Abstract

Molybdenum (Mo) microbial bioreduction is a phenomenon that is beginning to be recognized globally as a tool for the remediation of molybdenum toxicity. Molybdenum toxicity continues to be demonstrated in many animal models of spermatogenesis and oogenesis, particularly those of ruminants. The phenomenon has been reported for more than 100 years without a clear understanding of the reduction mechanism, indicating a clear gap in the scientific knowledge. This knowledge is not just fundamentally important—it is specifically important in applications for bioremediation measures and the sustainable recovery of metal from industrial or mine effluent. To date, about 52 molybdenum-reducing bacteria have been isolated globally. An increasing number of reports have also been published regarding the assimilation of other xenobiotics. This phenomenon is likely to be observed in current and future events in which the remediation of xenobiotics requires microorganisms capable of degrading or transforming multi-xenobiotics. This review aimed to comprehensively catalogue all of the characterizations of molybdenum-reducing microorganisms to date and identify future opportunities and improvements.

## 1. Introduction

Human activities are endangering the environment. Due to the growth of the population, intensive industrialization, in addition to urbanization and agriculture, has caused significant damage to the environment. The overexploitation of natural resources and ignorance of the laws of nature have exacerbated these problems. Pollution caused by hydrocarbons and metal ions has increased in recent years [1,2], representing a significant global problem.

Toxicity cases ranging from acute to chronic, within occupational and environmental high-exposure settings, have been identified to be caused by toxic agents from metals and their compounds. Typically, heavy metals naturally exist in the environment. Since the pre-industrial period, the level of heavy metals has significantly increased due to anthropogenic activities [3,4,5]. A considerable and indiscriminate release of pollutants into the environment has occurred in parallel with the growing population and intensity of industrialization [6,7]. Harmful effects on human health and biota can be exerted when heavy metals reach levels above the critical load [8,9,10,11]. In their elemental forms and different combinations, metals such arsenic, cadmium, chromium, cobalt, copper, lead, mercury, molybdenum, nickel, silver, and zinc are known to be toxic [12,13,14], and they are also non-degradable [15]. Thus, the accumulation of metals within the food chain can lead to a serious threat to the ecosystem [16] due to their carcinogenic and mutagenic properties [17]. Pollution caused by heavy metals has become a global public health concern. Hence, their removal from the environment is vital.

Molybdenum (Mo) is an important trace element that acts as a micronutrient that is necessary as a co-factor for more than 50 enzymes [18,19]. Within animal and plant physiology, for example, it helps to promote cellular function by catalyzing a variety of hydroxylation and redox transfer reactions [20]. Due to the heavy usage of molybdenum in the industrial manufacturing of ceramics, glass, contact lens solutions, metallurgical processes, lubricants, pigments, catalysts, electronic products, and color additives in cosmetics, the risks towards humans exposed to its toxicity have also arisen [18,20]. Increments up to 0.5 mg/L in the level of molybdenum have been discovered in the groundwater around mining areas, which is above the World Health Organization’s (WHO’s) recommended limit of 0.07 mg/L in drinking water [21]. Animals that have been in direct contact with molybdenum taken via the drinking of water or the foraging for plants are likely to portray symptoms of hypocuprosis or suffer from molybdenosis over a long exposure period [18].

For over a century, the microbial molybdate reduction to Mo-blue has not been understood. This phenomenon has been proven to be enzymatic rather than abiotic [22]. Previously, the focus of bacterial molybdate reduction research was on isolating molybdenum-reducers with higher Mo-blue production capacities. However, because most polluted sites contained mixed contaminants from organic and inorganic origins, effective remediation has thus become a complex task. During the past five years, attention has shifted towards isolating microorganisms with multi-reduction and/or degrading potential that could be used to remediate co-contaminated areas. Recently, several molybdenum-reducing bacteria with the potential to degrade other organic contaminants have been isolated. Therefore, a further understanding of the reduction mechanism and kinetics of Mo-reducing enzymes through various optimization processes will help in understanding the phenomenon of molybdate reduction to Mo-blue. In turn, this would be an important step towards the effective translation of laboratory findings to field practice.

## 2. Molybdenum (Mo)

Molybdenum is located in group VI, period V, of the transition series as a metallic element. The atomic number for this element is 42, and it has a relative atomic mass of 95.94 g/mol, with melting and boiling points of 2623 and 4639 °C, respectively. Because molybdenum metal does not freely exist in nature, it typically presents either as molybdenite (molybdenum disulfide: MoS_2_), wulfenite (lead molybdate), or powellite (calcium molybdate). The direct mining of molybdenite, which is commonly undertaken, enables the metal to be obtained. Furthermore, the metal can also be recovered as a byproduct of copper mining [23,24,25]. Molybdenum is commonly used in the industrial manufacturing of non-ferrous alloys, special steels, electrical contacts, X-ray tubes, spark plugs, tungsten production, glass-to-metal seals, and pigments. Due to several unique properties, molybdenum disulfide can be used as a lubricant additive, and molybdenum compounds are used in fertilizers or directly on seeds to mitigate molybdenum deficiency in crops [26,27].

Pure molybdenum naturally exists as a silvery-white metal with variable oxidation states between 2 and 6, the most stable of which are Mo^4+^ and Mo^6+^ [28]. Because molybdenum primarily exists as molybdate anions (MoO_4_^2−^) in nature, it can be combined to form an assortment of polymolybdate compounds [26,29,30]. Examples of these compounds that are soluble in an aqueous medium at room temperature are sodium molybdate (Na_2_MoO_4_), ammonium molybdate ((NH_4_)_2_MoO_4_), and ammonium paramolybdate ((NH_4_)_6_Mo_7_O_24_.4H_2_O). By comparison, molybdenum trioxide (MoO_3_) is sparingly soluble, and other compounds such as molybdenite (MoS_2_), calcium molybdate (CaMoO_4_), molybdenum chloride (MoCl_5_), and metallic molybdenum (Mo) are completely insoluble in an aqueous medium [26,28]. Molybdenum is one of the important trace elements needed by most living organisms in daily life processes [7]. Molybdenum is commonly present at the active site and plays a role as a cofactor to more than 50 enzymes involved in sulfur, nitrogen. and carbon cycles [18,19]; these enzymes include aldehyde oxidase, nitrogenase, sulfite oxidase, and xanthine oxidase. In addition, molybdenum acts as an agent in electron transport [19,28].

### 2.1. Molybdenum Entry Routes in Animals

Molybdenum and molybdenum compounds may enter the body by either the oral intake of food and drinking water or inhalation. Though absorption by the gastrointestinal tract is rapid and nearly complete, the efficiency and rate of absorption via inhalation are still unknown [28]. The solubility of the compounds and diet composition affect the rate of absorption following oral ingestion [28]. In addition, the existence of molybdenum in a chemical form significantly influences its rate of bioavailability, which is also dependent on the animal species [5,27,31,32,33]. A higher absorption capacity of hexavalent molybdenum (Mo^6+^), which is readily absorbed following oral administration, can be seen in non-ruminant animals compared to ruminant animals. In contrast, tetravalent molybdenum (Mo^4+^) has been found to not be readily absorbed [34]. Within the human gastrointestinal tract, the absorption of dietary molybdenum is estimated to be between 30% and 70% [35]. This absorption takes place rapidly within the blood circulation and organs, and it has a high potential to cross the placental barrier. The highest percentage of the absorption of molybdenum occurs in the bones, liver, and kidneys, and no apparent bioaccumulation of molybdenum has been reported in the human tissues [27,36]. Regarding animals, molybdenum compounds are mostly excreted among rodents via urine and, to a lesser extent, in feces [27,37]. Molybdenum excretion for cattle, sheep and horses, however, is generally shared between feces and urine because the absorption from the gastrointestinal tract is incomplete [27,34,37], whereas the intake and excretion of molybdenum are mostly balanced in most non-ruminants, including humans [36].

Animals suffering from copper deficiency have been indicated to be more susceptible to molybdenum toxicity, and the complex interaction between molybdenum with copper and sulphate has yet to be discovered. Non-ruminants can be given dietary sulfate to minimize the symptoms of molybdenum poisoning, whereas dietary sulfate administered to copper-deficient animals will exacerbate the problem [27,37].

### 2.2. Molybdenum Toxicity

Acute or chronic exposure can be used to evaluate the effects caused by molybdenum compounds. Data obtained on human toxicity as a result of chronic exposure are currently limited, and no relevant data are currently available on the acute toxicity of molybdenum compounds. Thus, the toxicity of these compounds needs to be assessed for different species of organisms because most of the chemicals are specified to their target and the effects may differ. The existence of numerous chemical forms of the molybdenum compounds should also be considered. The toxicity of molybdenum compounds in different animal models has been reported in several studies. Hence, it is important to identify the toxicity to spermatogenesis and oogenesis, particularly those of ruminants.

#### 2.2.1. Toxicity to Spermatogenesis and Oogenesis

In one study, the effects of dietary molybdenum on pup growth during lactation and the reproductive ability of Long–Evans rats fed for 13 weeks with diets containing a graded dose of molybdenum (0.1, 2, 8, or 14 mg/kg/day) and 5–20 mg/kg copper showed noticeable infertility among males due to the degeneration of the seminiferous tubules. Furthermore, in the groups that received the two highest doses, more pronounced effects were seen. Lactating mothers exposed to the two highest doses and their pups experienced lower weight-gain during lactation compared with those in the lower-dose groups [38]. The oral gavage of sodium molybdate with a dose greater than 30 mg/kg body weight [39] has been reported to cause a dose-dependent degeneration in testicular morphology and its function. This was observed from the decline in sperm motility, concentration, normal morphology and epididymides of the tested rats. Therefore, molybdenum can affect sperm quality by modulating complex testicular oxidative stress processes. For rats undergoing treatment with 12 mg/kg/day of tetrathiomolybdate for sixty days, a significant decrease in their epididymal weight, sperm motility, count, and morphologic abnormalities, in addition to histopathological alterations in the epididymis and testes, were observed [40]. Another report revealed similar results in which a decrease in the number of germ cells and mature spermatocytes was found in the testes of rabbits fed with carrots containing 39 mg Mo/kg dry weight, in addition to the appearance of degenerated cells and a large number of syncytial giant cells among the spermatogenic cells in the seminiferous tubules relative to the control [41]. The usage of doses of molybdenum greater than 100 mg/L resulted in a negative effect on mice testes (complete lack of libido and sterility), accompanied by alterations in the levels of malondialdehyde (MDA), superoxide dismutase (SOD), and glutathione peroxidase (GPx) [18].

#### 2.2.2. Mechanism of Molybdenum Toxicity in Ruminants

Molybdenum endorses cellular function by catalyzing a variety of hydroxylation and redox transfer reactions [18,19] that are essential within animal and plant physiology [20]. Molybdenum excretion for ruminants such as cattle, sheep, and horses is generally shared between faces and urine because the absorption from the gastrointestinal tract is incomplete [27,34,37]. In contrast, the intake and excretion of molybdenum for most non-ruminants, including humans, are usually balanced [36]. Copper-deficient animals are likely to be more susceptible to molybdenum toxicity than those on copper-adequate diets, as seen in the interaction between molybdenum, copper, and sulfate. Dietary sulfate can be administered for non-ruminants to avoid molybdenum poisoning, whereas dietary sulfate administered to copper-deficient animals could worsen the situation [27,34,37]. For ruminants, a molybdenum concentration of 10 mg/kg of body weight will result in tissue copper depletion, an effect that is enhanced by dietary sulfate [27,42].

One of the most important biological interrelationships between animal nutrition and medicine can be seen in the complex interaction between molybdenum, copper, and sulfate metabolisms regarding the kinetics, bioavailability, and utilization of copper. This interrelationship, however, is not yet fully understood. Among ruminants, different molybdenum compounds react with sulfides to form thiomolybdate compounds that then further react with copper to form an insoluble complex that is poorly absorbed. A decreased copper uptake impairs its utilization and results in the synthesis of several copper-dependent proteins [26,33,37,43]. Thus, a secondary copper deficiency is triggered by a reduced copper bioavailability. Copper excretion increases with the excessive intake of molybdenum. The administration of tetrathiomolybdate may aid in the treatment of chronic copper poisoning. The tolerance of monogastric animals to molybdenum toxicity is usually due to the inadequate production of thiomolybdates by the intestinal flora [34,37]. In addition, the functions of several enzymes involved in collagen and elastin stability, as well as their maintenance, can be impaired due to chronic exposure to molybdenum [44]. Therefore, this may be associated with the risks of cardiovascular disorders. Molybdenum exposure may also reduce phospholipid synthesis in nerve tissues within a similar vein, resulting in demyelination and neurologic abnormalities [27,37].

A primary manifestation of molybdenum toxicity can be presented in impaired copper utilization and metabolism resulting from a secondary copper deficiency (molybdenosis) [26,45,46]. This usually can be seen in at the scale of the herd, with a morbidity of up to 80% in cattle. Most cattle present with symptoms of persistent diarrhea associated with green liquid feces, which is often referred to as teart scours. In addition, the depigmentation of the coat is typically noticed among black animals as a consequence of impaired tyrosinase activity and reduced melanin synthesis [44]. A decline in bone mineralization due to the competition of molybdenum with phosphorous utilization causes microcytic hypochromic anemia, joint pain with associated lameness, and osteoporosis. Regarding heifers, decreased fertility, milk production, and weight loss at puberty are typically seen, whereas among sheep (particularly lambs less than 30 days old), enzootic ataxia or sway back syndrome present as stiffness of the legs and back, respectively, and difficulty standing is seen [47]. Within one-to-two weeks of chronic exposure to molybdenum, the affected animals usually exhibit the abnormal development of connective tissue and growth. Because acute toxicity may occasionally be encountered in cattle or sheep, the symptoms of anorexia and lethargy may be witnessed within three days, and deaths can commence within the first week [27,45].

### 2.3. Bioremediation

In recent years, the bacterial-based remediation of environmental pollution has drawn significant interest as a promising innovative technology [48,49,50]. In order to abolish, attenuate, or transform pollutants into less hazardous products in combination with carbon dioxide, water, inorganic salts, and microbial biomass production, the unique catabolic property of microorganisms has been employed [51]. However, inorganic toxicants, e.g., heavy metals, are not degraded by microorganisms. Therefore, biologically encoded changes in the redox state and active metabolic capacity of the microbe are needed. In general, heavy metals are essentially needed by most microorganisms for life processes to occur. However, the adsorption capacity depends on both total microbial biomass and the system’s geochemistry. A number of metals’ oxyanions do not interact with microorganisms via assimilation, and so their bioremoval is focused on enzyme-catalyzed redox conversion to their precipitable forms [7].

#### 2.3.1. Molybdenum Pollution

The diversity of molybdenum, which is used in superalloys, nickel-base alloys, lubricants, additives, glassworks, paint, pigments, electronics, and many other uses, has been proven to be valuable. Molybdenum can be present in the discharged effluent associated with these products [52]. Due to decades of heavy industrialization, heavy metal pollution in Tokyo Bay and the Black Sea, including that of molybdenum, has reached concerning levels [52]. In Tyrol, Austria, molybdenum in the soil used to feed ruminants can exceed 200 ppm, which resulted from decades of industrial exhaust discharge and has led to deaths and scouring [53]. A light-bulb plant built in the early 1970s in China has resulted in soil molybdenum pollution of 0.25–252 mg/kg [21]. Unusually high concentrations of Mo and Nb were found in the atmosphere of Islamabad, Pakistan, and the elevated levels of these elements were suggested to have originated from anthropogenic activities [54]. In Jordan, areas exposed to extensive oil-shale exploration and intensive agricultural activities have been found to be subject to molybdenum pollution of 11.7 mg/kg of soil [55].

In addition to industrial pollution, mining sites are a major cause of molybdenum pollution. Molybdenum is the main dissolved metal in the aqueous effluent from many uranium flotation mills, probably due to the high solubility of the molybdenum metal in the form of molybdate ions at the high pH values that are used, whereas most other metals are precipitated as hydroxides. For instance, in New Mexico, molybdenum and other toxic metals released from the Molycorp molybdenum mine resulted in levels of these metals exceeding the New Mexico environmental standard in the Red River and caused the extinction of many of the river’s floras and fauna [56]. A complex tailings dam was found to contain molybdenum in the local region of the Miduk Copper Complex copper mine in Iran, where molybdenum is a byproduct [57]. Geochemical approaches have been used to analyze the speciation and levels of Mo, in addition to Zn, Cu, and Fe, in Nver River sediments polluted by Mo mining in western Liaoning, Northeast China. Metal characterization studied using X-ray fluorescence and X-ray diffraction has shown that the quantity of Mo approaches 1127.9 times the concentration of Mo in the Earth’s crust. This analysis revealed that Mo tailings ponds may have a close association with the high sediment amounts of these metals, and Mo in sediment may pose a high danger to the surrounding ecosystem [58].

In Armenia, nearly 300 square kilometers of land are polluted with heavy metals such as lead, copper, and molybdenum from the Alaverdi copper–molybdenum mine in the area. Land adjacent to the mining activities suffers from the worst pollution, with levels of these heavy metals at 20–40 times the limit [59]. The concentration of dissolved Mo has been estimated to be as high as 900 ppm in the U.S.A. from a uranium mill in southern Colorado. Dissolved Mo exists in amounts up to around 25 ppm in the aqueous discharge from major molybdenum mills also located in Colorado [60]. One of the highest documented levels of molybdenum pollution in soils or water bodies is approximately 20.8 mM, or 2000 ppm [61]. In 2007 and 2008, a series of mysterious cow deaths occurred across the dark brown soil zone of Saskatchewan during the calving season, causing the deaths of 46 cows for 11 cow producers. Molybdenum toxicity was identified to be the major reason for the loss [62]. Molybdenum can be presented in the form of molybdenite, a byproduct of gold and copper mining. In recent years, the contamination of several thousand acres of paddy land occurred due to episodic pollution cases involving leakage from mining sites [63]. Furthermore, the main hidden source of molybdenum pollution in Malaysia is via the indiscriminate release of lubricant oil wastes, which contain 1–5% Mo, with the highest molybdenum concentration of 17.86 mg per kg soil found at one location [64].

#### 2.3.2. Molybdenum Bioremediation

Microorganisms utilize the mechanisms of bioreduction, bioprecipitation, bioaccumulation or sequestration, efflux pumping, and biosorption to detoxify and immobilize metal ions of molybdenum, chromium, copper, and mercury [12,13,65,66,67,68]. During the past century, microbial molybdate (Mo^6+^) reduction was first reported in *Escherichia coli* by Capaldi and Proskauer [69]. However, the details of the reduction phenomenon were only discovered during the last three decades by Campbell et al. in *E. coli* K12 [70]. The work of [71] on *Thiobacillus ferrooxidans* (now *Acidithiobacillus ferrooxidans*) was then continued on *Enterobacter cloacae* strain 48 or EC 48 [72] and other Mo-reducing bacteria (Table 1). Molybdenum can also be reduced to a lower oxidation state by the action of sulfide, produced by sulphate-reducing bacteria such as *Desulfovibrio desulphuricans* and *Desulfovibrio vulgaris*, where molybdenum is reduced to MoS_2_ (Mo^4+^). Molybdenum disulfide is insoluble and precipitates out of solutions, which is another avenue for molybdenum bioremediation [73].

The earliest documented report on molybdenum bioremediation was in Tyrol, Austria. A plant-microbial consortium was used to treat large pasture areas that had been contaminated with molybdenum from industrial discharge. Soluble toxic molybdenum was detoxified using microbes from sewage, which transformed soluble molybdenum into insoluble compounds [53]. The use of molybdenum-reducing microorganisms in this case, as well as in the future using currently isolated molybdenum-reducing bacteria [74,75,76,77,78,79,80,81,82], represents a sustainable means of remediating molybdenum pollution.

#### 2.3.3. Mechanism of Molybdenum Reduction to Mo-Blue

The mechanism of microbial molybdate reduction to Mo-blue was first proposed for *E. cloacae* strain 48 [72,83]. Enzyme-catalyzed redox was involved in the change in the oxidation state of molybdenum (M^6+^ to M^5+^) before phosphate joined to form Mo-blue. Similar Mo-blue spectra were produced in both the bacterial molybdate reduction and ascorbate-reduce phosphate determination methods, indicating the possible formation of the phosphomolybdate intermediate [84]. At this stage, the involvement of the phosphomolybdate species was first suggested as occurring under static growth conditions and high cell concentrations in a shaking mode of growth, and semi-anaerobic conditions may occur. Hence, most heterotrophs undergo fermentation and release organic acids. Consequently, the pH of the medium is lowered and molybdate ions are converted to phosphomolybdate [85,86].

**Figure 1:**
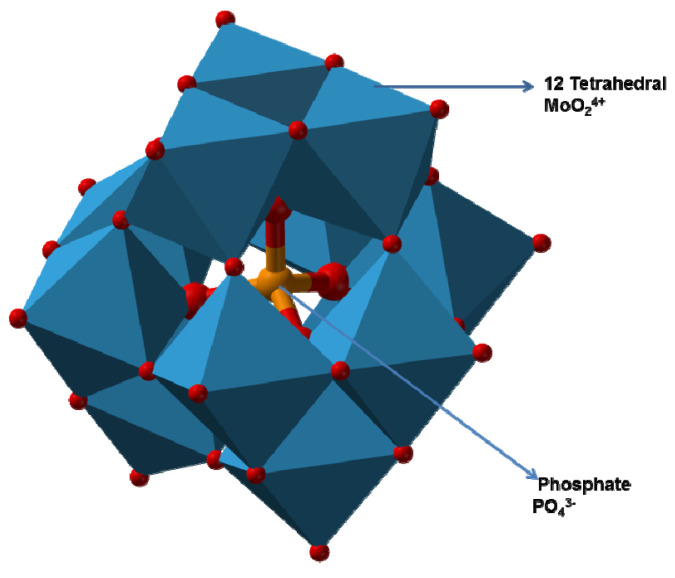
A typical 12-phosphomolybdate structure based on the phosphotungstate structure. The image is under a Creative Commons license. The phosphotungstate structure: ‘Phosphotungstate-3D-polyhedra’ image was created by Ben Mills (benjah-bmm27). This image is in the public domain. Source: https://en.wikipedia.org/wiki/File:Phosphotungstate-3D-polyhedra.png (accessed on 12th of November 2020)

In an aqueous medium (pH 7), molybdenum exists as molybdate ions (MoO_2_^4+^). Molybdate ions, however, form a unique complex structure called polymolybdate anions in an acidic solution. This is a hallmark of molybdenum chemistry. The polymolybdate ions then further combine with other anions, such as sulfate, phosphate, or silicate, to form a mega-complex (Figure 1), the so-called heteropolymolybdate (phosphomolybdate, silicomolybdate, and sulfurmolybdate) [85,87]. This phenomenon is believed to be the first to occur during molybdate reduction to Mo-blue. Reducing agents, such as dithionite, ferrous ions, and stannous ions, can donate two electrons to the formed heteropolymolybdate (PMo_12_O^3+^: 12-molybdophosphate), hence converting it to molybdenum blue. Both of these donated electrons are spread across the entire polymetallate sphere by a thermally-activated hopping process, as shown in electron spin resonance (ESR). The two reduced electrons are in constant motion, as shown by nuclear magnetic spectroscopy, and therefore account for the valency of all of the twelve molybdenum atoms. The movement of electrons across the Keggin complex causes a mixed valency of molybdenum oxidation states, is between 6+ and 5+ [88,89].

**Figure 2:**
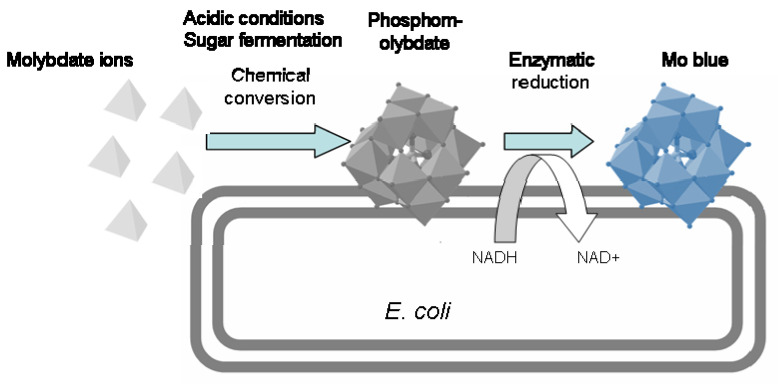
A schematic representation of the probable mechanism of molybdate reduction to molybdenum (Mo)-blue.

The spectral scanning of Mo-blue produced by all isolated molybdenum-reducing bacteria is remarkably similar to the ascorbate-reduced phosphate determination method. In the phosphate determination method, the Mo-blue spectrum produces a characteristic peak at 890 nm and a shoulder at 710 nm, whereas the bacterial spectrum generally shows a peak at 865 nm and a shoulder at 700 nm [86]. Cell contact is required in molybdate reduction to Mo-blue, as reported in [70], and phosphomolybdate has been suggested as a possible identity of Mo-blue. Because there are a number of lacunary species of phosphomolybdate that can change, as well as due to changes in pH, it is hard to differentiate between the species formed during bacterial molybdate reduction. Spectral absorbance scanning has therefore been suggested as an adequate method to differentiate between the major heteropolymolybdates, such as phosphomolybdate, sulphomolybdate, and silicomolybdate [87]. Based on the similar absorption spectrum to that of Mo-blue using the ascorbic acid method and a reduction in pH during fermentation of most Mo-reducing bacteria, Shukor et al. proposed [86] that phosphomolybdate is an intermediate during the bacterial reduction of molybdenum to Mo-blue and that this phosphomolybdate is chemically formed (Figure 2). Furthermore, entrapping *Enterobacter cloacae* 48 in a dialysis tube [90] provided evidence to support the earlier suggestion of Campbell that Mo-blue production in microorganisms requires cell contact [29,70].

### 2.4. Characteristics of Previously Isolated Molybdenum-Reducing Bacteria

To date, the molybdenum-reducing bacteria are known to come from twelve different genera (*Acinetobacter*, *Bacillus*, *Burkholderia*, *Enterobacter*, *Escherichia*, *Klebsiella*, *Pseudomonas*, *Pantoea*, *Morganella*, *Clostridium*, *Raoultella*, and *Serratia*) isolated from different soils and polluted waters around the world. From the isolated molybdenum-reducing bacteria (52 bacteria), 45% are from Malaysia (23 bacteria), followed by Nigeria (eight bacteria), Egypt (seven bacteria), and Indonesia (five bacteria). The remainder are from Sudan (two bacteria) and Pakistan (two bacteria), as well as Iraq, China, USA, and Antarctica, at one bacterium each (Table 1). *E. coli* K12 was the only standard ATCC-type strain (strain ATCC 10798). A molybdenum-tolerant bacterium (*Bacillus subtilis* LM 4-2) from China was isolated [91] from a molybdenum mine but was not reported to reduce molybdenum to molybdenum blue. Though a complete genome sequence of this molybdenum-resistant bacterium has been reported, the characterization work was excluded. Therefore, a new research direction has emerged in the genome annotation of molybdenum-reducing bacteria, particularly for sequencing work of the isolate. Of the twelve genera that are currently known for molybdenum reduction, the genera *Bacillus* spp. and *Staphylococcus* spp. are gram-positive rod and cocci, respectively. The remaining genera are gram-negative rods, with the majority belonging to the Enterobacteriaceae family (Table 1). For 80% of the isolated reducers, the optimum temperature that supports Mo-blue production was found to be between 30 and 37 °C; for 10%, the temperature was between 25 and 30 °C [67,92]; for 5%, it was between 15 and 20 °C [93]; and for 5%, it was 40 °C [94]. Of these, one was a psychrotolerant bacterium isolated from Antarctica, and two were thermophilic bacteria isolated from Egypt (Table 1). The molybdate reduction was discovered to be the most favorable at pH values between 5 and 6 [70], 5.5 and 6.8 [30,66,95,96,97,98,99], 7 and 8 [67,72,92,95,100,101,102,103], and 6.5 and 7.5 [93].

An estimated 80% of the Mo-reducers require glucose as the best electron donor source for optimum Mo-blue production [30,66,67,68,70,92,93,94,96,97,98,99,100,103,104], 20% require sucrose [72,90,95,101], and the remaining 5% require fructose [102]. These requirements are important for easily assimilable carbon sources for most of the Mo-reducers, thus indicating that molybdenum reduction is a growth-associated process.

As a general observation, molybdate reduction to molybdenum blue is inhibited at phosphate concentrations above 5 mM, which might be due to the physical interaction with phosphomolybdate substrate rather than an enzymatic inhibition [29]. Nearly all Mo-reducers achieve the optimal Mo-blue production at phosphate concentrations of between 2 and 5 mM. However, *Pseudomonas putida* strain Amr-12, *Klebsiella oxytoca* strain Aft-7, *Bacillus amyloliquefaciens* strain Neni-9, *Bacillus amyloliquefaciens*, *Enterobacter cloacae*, and *Bacillus* sp. strain khayat achieve their optimum at phosphate concentrations between 5 and 7.5 mM, whereas the optimum of *Pseudomonas* *aeruginosa* strain Amr-11 is between 2.5 and 7.5 mM (Table 1).

Several other molybdenum-reducing bacteria, e.g., *Klebsiella oxytoca* strain Hkeem and *Escherichia coli* K12, can also tolerate and support optimal Mo-blue production at molybdate concentrations as high as 80 mM. Generally, most Mo-reducing bacteria exhibit an optimum reduction from 25 to 55 mM sodium molybdate (Table 1). In contrast, *Bacillus* sp. strain khayat, *Klebsiella oxytoca* strain Saw-5, *P. aeruginosa* strain Amr-11, *Klebsiella oxytoca* strain Aft-7, *Pseudomonas putida* strain Amr-12, *Pseudomonas* sp. strain DRY2, *Acinetobacter calcoaceticus* strain Dr.Y12, *Serratia marcescens* strain Dr.Y9, *Serratia marcescens* strain DRY6, and *Enterobacter cloacae* strain 48 require lower molybdate concentrations of between 5 and 20 mM. The highest is for *Pseudomonas* sp. isolated from Nigerian soil, which has an optimum at 100 mM (Table 1). This is helpful for molybdenum reducers that can tolerate and reduce the concentrations that are higher than 20 mM, because molybdenum pollution in a previous report reached as high as 2000 ppm (20.8 mM molybdate) [61].

Therefore, almost all of the Mo-reducers are similarly inhibited by the respiratory inhibitors and other toxic metals, such as silver, arsenic, cadmium, chromium, copper, lead, mercury, and zinc [30,90,93,98].

It has been reported that nearly all of the molybdenum reducers are susceptible to similar toxic heavy metals reported in this work (Table 1). In the chromate-reducing bacteria *Bacillus* sp. [105] and *Enterobacter cloacae* strain H01 [106], toxic cationic metals, such as mercury and copper, strongly inhibit bacterial chromate reduction and pose a threat to the remediation of anionic metals such as molybdate, chromate, and vanadate. These toxic cationic metal ions bind to the sulfhydryl, carboxyl, amide, phosphoryl, and amine groups of many housekeeping enzymes, including metal reductases, ultimately halting remediation. A strategy to combat this is the addition of nontoxic cationic metals’ chelating or reacting compounds, such as manganese oxide, phosphate, calcium carbonate, and magnesium hydroxide, which react and reduce the solubility of cationic metals, thus allowing for the commencement of bioremediation [107,108]. Another alternative, specifically for molybdenum, is the immobilization of the Mo-reducing bacterium in an entrapment agent such as dialysis tubing, which has been demonstrated to offer protection to the toxicity of the co-presence cationic metal ions [109].

**Table 1:** Characteristics molybdenum-reducing bacteria.

Bacteria	Specialization of the Bacteria	Optimal pH and Temperature	Preferred Carbon Source	MoO_4_ (mM)	PoO_4_ (mM)	Heavy Metal Inhibition	1° Model and Kinetics of Reduction	Optimization Method	Author
*Bacillus amyloliquefaciens* strain Neni-9 (Indonesia)	Mo reduction Growth on carbaryl and carbofuran	pH 6.3 and 6.5, 30–37 °C	glucose	20–30	5.0–7.5	Ag^+^, Cr^6+^, Cu^2+^ Hg^2+^	1° model Mo reduction model using modified Gompertz	OFAT	[80]
*Pseudomonas* sp. (Nigeria)	Mo reduction	pH 6.5–7.5 37 °C	glucose	100	3.5–7.5	n.a.	n.a.	OFAT	[81]
*Pantoea* sp. strain HMY-P4 (Nigeria)	Mo reduction	pH 6.0–8.0 35–40 °C	glucose	20–40	5.0	n.a.	Aiba; *q_max_, K_s_*, and *K_i_* of 0.89 μmol Mo-blue per h, 5.84 mM, and 32.23 mM, respectively	OFAT	[76,110]
*Enterobacter cloacae* (Nigeria)	Mo reduction	pH 6.5–7.0 35–40 °C	glucose	80–100	5.0–7.5	n.a.	Monod; *q_max_* and *K_s_* of 2.77 μmole Mo-blue h^−1^, and 12.42 mM, respectively	OFAT	[77,111]
*Morganella* sp. (Nigeria)	Mo reduction	pH 6.0–7.5 35 °C	glucose	40	3.5	n.a.	Teissier-Edward; *q_max_, K_s_*, and *K_i_* of 7.77 mmole Mo-blue h^−1^, 26.63 mM, and 51.39 mM, respectively.	OFAT	[78]
*Pseudomonas.* strain Dr. Y Kertih(Malaysia)	Mo reduction Growth on phenol, acrylamide, nicotinamide, acetamide, iodoacetamide, propionamide, acetamide, sodium dodecyl sulfate (SDS), and diesel	pH 6.0–6.3 25–40 °C.	glucose	20	5.0	Ag^+^, Pb^2+^, As^5+^ Hg^2+^	n.a.	OFAT	[112]
*Clostridium pasteurianum* BC1 (USA)	metallic (Mo0) nanoparticles 5–20 nm in size Degradation of methyl orange	pH 6.8 n.a.	peptone	20.67	1.74			OFAT	[113]
microbial electrolysis cells consortium (China)	Mo reduction, Tungsten reduction and acetate biodegradation Hydrogen production	pH 3.0 22 °C	acetate	1	n.a.	n.a.	n.a.	OFAT	[75]
*Raoultella ornithinolytica* strain Mo1 (Egypt)	Mo reduction	pH 6, 30 °C	glucose	20		n.a.	n.a.	OFAT	[114]
*Raoultella planticola* strain MoI (Iraq)	Mo reduction	pH 6 30 °C	glucose	20		n.a.	n.a.	OFAT	[114]
*Bacillus sonorensis* strain Pharon3 (MK078035) (Egypt)	Mo reduction Thermophilic bacterium	pH 7.07 52.2 °C	glucose	10	4.0		n.a.	RSM (CCD)	[82]
*Bacillus tequilensis* strain Pharon2 (MK078034) (Egypt)	Mo reduction Thermophilic bacterium	pH 7.02 46.1 °C	sucrose	10	4.0		n.a.	RSM (CCD)	[82]
*Bacillus* sp. strain Neni-12 (Indonesia)	Mo reduction Growth on coumaphos	pH 6.3 25–37 °C	glucose	15–20	5.0	Ag^+^, Cr^6+^, Hg^2+^	1° model, coumaphos growth model using modified Gompertz	OFAT	[79]
*Pseudomonas* sp. (Nigeria)	Mo reduction	pH 6.5–7.0 35– 40 °C	glucose	40–60	3.5	n.a.	n.a.	OFAT	[115]
*Burkholderia vietnamiensis* AQ5-12 (Malaysia)	Mo reduction Glyphosate degradation	pH 6.25–8.0 30–40 °C	glucose	40–60	5.0	n.a.	n.a.	OFAT	[116]
*Burkholderia* sp. AQ5-13 (Malaysia)	Mo reduction Glyphosate degradation	pH 6.25–8.0 35–40 °C	glucose	40–50	5.0	n.a.	n.a.	OFAT	[116]
*Serratia marcescens* strain KIK-1 (Nigeria)	Mo reduction Decolorization of various azo and triphenyl methane dyes	pH 5.8–6.5 34–37 °C	glucose	10–25	5.0	Ag^+^, Cr^6+^, Hg^2+^, Cu^2+^	n.a.	OFAT	[117]
*Pseudomonas putida* strain Egypt-15 (Egypt)	Mo reduction Growth on PEG 4000	pH 6.5 34 °C	glucose	20	5.0	n.a.	1° model, PEG 4000 growth model using modified Gompertz	OFAT	[74]
*Bacillus amyloliquefaciens* (Malaysia)	SDS degradation	pH 5.8–6.3 25–34 °C	glucose	30–50	5.0–7.5	Hg^2+^, Cu^2+^, Ag^+^			[118]
*Serratia* sp. strain HMY1 (Nigeria)	Mo reduction Cyanide degradation Q10 value of 2.038 and a theta value of 1.08	pH 6.5–7.0 30–35 °C	sucrose	55	3.95	n.a.	1° model, Mo reduction model using modified Gompertz	RSM (CCD)	[119,120,121]
*Enterobacter* sp. Strain Saw-2	Mo reduction Growth on phenol and catechol	pH 6.3–6.8 34–37 °C	glucose	15–30	5.0	n.a.	n.a.	OFAT	[122]
*Serratia* sp. strain HMY3 (Nigeria)	Mo reduction Cyanide degradation	pH 6.5 35 °C	sucrose	55–57.5	3.95	As^3+^, Cr^6+^, Hg^2+^, Cu^2^	Luong; *q_max_*, *K_s_*, *S_m_*, and *n* were 25.32 h^−1^, 113.4 mM, 55.43 mM, and 1.42, respectively.	OFAT	[123]
*Bacillus* sp. strain Neni-10 (Indonesia)	Mo reduction Decolourisation of the dye Metanil Yellow	pH 6.3 34 °C	glucose	20	2.5–7.5	Ag^+^, Cu^2+^, Cr^6+^, Hg^2+^	1° model Mo reduction best model using Baranyi–Roberts	OFAT	[124,125]
*Pseudomonas* sp. strain 135 (Malaysia)	Mo reduction, Growth on acrylamide, acetamide, and propionamide acrylamide can support Mo reduction	pH 6.0–6.3 25–40 °C	glucose	15–25	5.0–7.5	Ag^+^, Cu^2+^, Cd^2+^, Hg^2+^	n.a.	OFAT	[126]
*Serratia marcescens* strain DR.Y10 (Malaysia)	Mo reduction Growth on acrylamide, propionamide, and acetamide	pH 6.0–6.5 30–37 °C	glucose	10–30	5.0	Ag^+^, Cu^2+^, Cr^6+^, Hg^2+^	n.a.	OFAT	[127]
*Pseudomonas aeruginosa* strain KIK-11 (Malaysia)	Growth on diesel and sodium dodecyl sulphate	pH 5.8–6.0 25–34 °C	glucose	30–40	5.0–7.5	Ag^+^, Cu^2+^, Hg^2+^	n.a.	OFAT	[128]
*Serratia* sp. *strain* MIE2 (Malaysia)	Mo reduction	pH 6.0 27 to 35^o^C	sucrose	20	3.95	Hg^2+^, Zn^2+^, Cu^2^	Teissier-Edward’s *q_max_,* and *K_s_* and *K_i_* 0.89 mmole Mo-blue h^−1^, 5.84 mM, and 32.23 mM, respectively	RSM (Box-Behnken and CCD)	[129,130]
*Bacillus* sp. strain khayat (Malaysia)	Mo reduction SDS Diesel-degrading	pH 5.8–6.8 34 °C	glucose	10–20	5–7.5	Ag^+^, As^3+^, Pb^2+^, Hg^2+^, Cu^2+^	n.a.	OFAT	[98]
*Burkholderia* sp. strain Neni-11 (Indonesia)	Mo reduction Amide-degrading	pH 6.0–6.3 30–37 °C	glucose	15	5	Ag^+^, Cr^6+^, Hg^2+^	1° model, Mo reduction model using modified Gompertz	OFAT	[131]
*Enterobacter* sp. strain Aft-3 (Pakistan)	Mo reduction Azo dye-decolorizing	pH 5.8–6.5 37 °C	glucose	20–25	5	Ag^+^, Cu^2^, Hg^2+^	n.a.	OFAT	[132]
*Klebsiella oxytoca* strain Saw-5 (Malaysia)	Mo reduction Glyphosate degradation	pH 6.3–6.8 34 °C	glucose	20–30	5	Ag^+^, Cd^2+^, Cr^6+^, Hg^2+^, Cu^2+^	n.a.	OFAT	[99]
*P. aeruginosa* strain Amr-11 (Egypt)	Mo reduction Phenol degradation	pH 6.3–6.8 34 °C	glucose	20–30	2.5–7.5	Ag^+^, As^3+^, Pb^2+^, Cd^2+^, Cr^6+^, Hg^2+^, Cu^2+^	n.a.	OFAT	[104]
*Klebsiella oxytoca* strain Aft-7 (Pakistan)	Mo reduction SDS degradation	pH 5.8–6.3 25–34 °C	glucose	5–20	5–7.5	Ag^+^, As^3+^, Pb^2+^, Cd^2+^, Cr^6+^, Hg^2+^, Cu^2+^	n.a.	OFAT	[97]
*Enterobacter* sp. strain Zeid-6 (Sudan)	Mo reduction Orange G decolorization	pH 5.5–8.0 30–37 °C	glucose	20	5	Ag^+^, Pb^2+^, Hg^2+^, Cu^2+^,	n.a.	OFAT	[92]
*Pseudomonas putida* strain Amr-12 (Egypt)	Mo reduction, Phenol and catechol degradation	pH 6.0–7.0 20–30 °C	glucose	20–30	5.0–7.5	Ag^+^, Cr^6+^, Hg^2+^	n.a.	OFAT	[30]
*Enterobacter* sp. Strain Neni-13	Mo reduction Growth on SDS	pH 6.0–6.5 37 °C	glucose	15	2.5–5.0	Ag^+^, Cd^2+^, Hg^2+^, Cu^2+^	1° model, SDS growth model using modified Gompertz	OFAT	[133]
*Bacillus* sp. strain Zeid 14	Mo reduction Growth on amides, acetonitrile, and acrylamide can support Mo reduction	pH 6.0–6.8 25–34 °C	glucose	10–20	5.0–7.5	Ag^+^, Cd^2+^, Cr^6+^, Hg^2+^, Cu^2+^	1° model, Mo reduction model using modified Gompertz	OFAT	[134]
*Klebsiella oxytoca* strain DRY14 (Malaysia)	Mo reduction SDS degradation	pH 7.0 25 °C	glucose	25–30	5	Ag^+^, Pb^2+^, Cd^2+^, Cr^6+^, Hg^2+^, Cu^2+^	n.a.	OFAT	[67]
*Bacillus pumilus* strain lbna (Malaysia)	Mo reduction	pH 7.0–8.0 37 °C	glucose	40	2.5–5	As^3+^, Pb^2+^, Zn^2+^, Cd^2+^, Cr^6+^, Hg^2+^, Cu^2+^	Luong, *q_max_, K_s_, S_m_*, and *n* values of 27.3 μmol Mo-blue h^−1^, 115.8 mM, 57.83 mM, and 1.405, respectively	OFAT	[135]
*Bacillus* sp. strain A.rzi (Malaysia)	Mo reduction	pH 7.3 28–30 °C	glucose	50	4	Cd^2+^, Cr^6+^, Cu^2+^, Ag^+^, Pb^2+^, Hg^2+,^ Co^2+^, Zn^2+^	Luong, *q_max_, K_s_, S_m_*, and *n* values of 5.88 mole Mo-blue h^−1^, 70.36 mM, 108.22 mM, and 0.74, respectively	OFAT	[68]
*Pseudomonas* sp. strain DRY1 (Antarctica)	Mo reduction	pH 6.5–7.5 15–20 °C	glucose	30–50	5	Cd^2+^, Cr^6+^, Cu^2+^, Ag^+^, Pb^2+^, Hg^2+^	n.a.	OFAT	[93]
*Klebsiella oxytoca* strain Hkeem (Malaysia)	Mo reduction	pH 7.3 30 °C	fructose	80	4.5	Cu^2+^, Ag^+^, Hg^2+^	n.a.		[102]
*Pseudomonas* sp. strain DRY2 (Malaysia)	Mo reduction	pH 6.0 40 °C	glucose	15–20	5	Cr^6+^, Cu^2+^, Pb^2+^, Hg^2+^	n.a.	OFAT	[94]
*Acinetobacter calcoaceticus* strain Dr.Y12 (Malaysia)	Mo reduction	pH 6.5 37 °C	glucose	20	5	Cd^2+^, Cr^6+^, Cu^2+^, Pb^2+^, Hg^2+^	n.a.	OFAT	[96]
*Enterobacter* sp. strain Dr.Y13 (Malaysia)	Mo reduction	pH 6.5 37 °C	glucose	25–50	5	Cr^6+^, Cd^2+^, Cu^2+^, Ag^+^, Hg^2+^	n.a.	OFAT	[66]
*S. marcescens* strain Dr.Y9 (Malaysia)	Mo reduction	pH 7.0 37 °C	sucrose	20	5	Cr^6+^, Cu^2+^, Ag^+^, Hg^2+^	n.a.	OFAT	[101]
*Serratia* sp. strain Dr.Y8 (Malaysia)	Mo reduction	pH 6.0 37 °C	sucrose	50	5	Cr^6+^, Cu^2+^, Ag^+^, Hg^2+^	n.a.	OFAT	[90]
*Serratia* sp. strain Dr.Y5 (Malaysia)	Mo reduction Purification of 1st Mo-reducing enzyme	pH 7.0 37 °C	sucrose	30	5	Cu^2+^	1° model Mo reduction best model using Huang model	OFAT	[100,136,137,138,139]
*Serratia marcescens* strain DRY6 (Malaysia)	Mo reduction	pH 7.0 35 °C	sucrose	15–25	5	Cr^6+^, Cu^2+^, Hg^2+^	n.a.	OFAT	[95]
*Enterobacter cloacae* strain 48 (Malaysia)	Mo reduction	pH 7.0 30 °C	sucrose	20	2.9	Cr^6+^, Cu^2+^	n.a.	OFAT	[72]
*Escherichia coli* K12	Mo reduction	pH 7.0 30–36 °C	glucose	80	5	Cr^6+^	n.a.	OFAT	[70]

Note: OFAT = one-factor-at-a-time; RSM = response surface method; CCD = central composite design.

### 2.5. Statistical-Based Optimization Compared to One-at-a-Time Approach

Statistical-based optimization, such as the response surface method (RSM), is a composition of statistical and mathematical techniques for empirically building a model. It is a well-known and current approach for constructing approximation models based on experimental observations [140,141,142]. A newer approach is the use of artificial neural networks (ANNs). Both the RSM and ANNs have individual advantages and disadvantages [143]. The RSM is, however, more commonly reported and hence allows for a good comparison with established RSM data. The RSM was developed by Box and Wilson (1951) and uses a meticulous arrangement of experiments with the ultimate objective of maximizing an answer (output variable). Multiple independent variables influence the performance (input variables). The use of the RSM based on a central composite design (CCD) to optimize molybdenum reduction has been carried out in bacteria such as *Serratia* sp. strain MIE2 [130], *Serratia* sp. strain HMY1 [121], *Bacillus sonorensis* strain Pharon3 (MK078035), and *Bacillus tequilensis* strain Pharon2 (MK078034) [82], and it has successfully improved the process compared to the one-factor-at-a-time (OFAT) method.

### 2.6. Mathematical Modelling of Molybdenum Reduction Profile and Kinetics

Bacterial growth-linked processes often demonstrate a unique phase. The specific growth rate commences at a value of zero, after which it accelerates to a maximal value (*μ_max_*) in a certain period, thus producing a lag time (λ). Within the growth curves, a final phase is involved in which the rate diminishes and ultimately reaches zero. Therefore, an asymptote (*A*) is obtained. Changes in the growth rate frequently result in a sigmoidal curve with the characteristic lag phase just after t = 0. An exponential phase then follows, and it is succeeded by a stationary phase and, finally, the death phase [144]. The lag period seen in the sigmoid shape has been attributed to the bacterial cells gearing their growth mechanism to adjust to a new environment of a vegetative state, particularly during storage. This adjustment period is commonly described as the lag period and can be described as a transient period that connects two autonomous systems. This introduction of the lag time or parameter is significantly less problematic than a mechanistic interpretation [145]. At the initial inoculation, it is presumed that each bacterial cell has different rates of growth. A nonlinear distribution can be illustrated if the rates of growth have been measured, as suggested by several studies [145,146].

In addition to the asymptotic value and the lag period, another valuable parameter of a growth curve is the maximum specific growth rate (*μ_m_*). This is commonly used in the development of secondary models, such as of the effects of substrate, product, pH, and temperature on the growth rate of the organism [144]. The logarithm of the activity or cellular microbial number, *μ_m_*, can be demonstrated by the slope of the line when the activity or organisms grow or expand exponentially [147]. The parameter is measured through manual estimation from the part of the curve that is approximately linear, and a linear regression is used to determine the slope of this curve. In practice, the linearization of the sigmoidal curve using natural logarithmic data transformation is one of the most frequently used methods, and it has been used to determine the specific reduction rate for molybdenum reduction in *Bacillus* sp. strain A.rzi [68]. In addition, the entire set of data can also be illustrated by a nonlinear regression growth model, which then estimates *μ_max_*, λ and A from the model itself [148]. 

The molybdenum reduction to molybdenum blue is a detoxification process, whereas the production of Mo-blue is a growth-associated process [66,67,90,94,95,96,102,109,131,149,150]. Various approaches to reduction modeling have been previously used [151], including logistic [144,152], Gompertz [144,153], Richards [144,154], Schnute [144], Baranyi–Roberts [155], von Bertalanffy [156,157], Buchanan three-phase [158], and the Huang model, which was used most recently [159] (Table 1) to model Mo-blue production from bacteria. It should be noted that the maximum specific growth rate or *μ_m_* was modified to *q_m_* and represents the maximum specific reduction rate. It appears that the modified Gompertz model is the best model to fit Mo-reduction in many cases (Table 2).

Once the maximum specific reduction rate values are obtained from the primary modelling exercise, the values can be used to model the effect of the substrate (molybdate) on the rate of reduction. Several important parameters, such as the specific reduction rate, theoretical maximum reduction, and determination of the reduction at high molybdenum concentrations (which affects the lag period of reduction and the inhibitory property of the substrate, i.e., molybdenum to Mo-blue production), can be shown by the mathematical modeling of the reduction process [151]. In addition, the specific reduction rate can be used in secondary modeling via models such as those of Haldane, Monod, Teissier, Aiba, Yano, and Luong. Though the use of these approaches in the modeling of reduction kinetics involving metal-reducing microbial processes is seldom studied, a similar implementation has been undertaken in the research of organic compound biodegradation. Haldane-type inhibition has been reported in scientific studies of the modeling of the reduction kinetics of heavy metals, including mercury [160], arsenate [161], and chromate [162], whereas a Monod kinetic approach was reported [163] for uranium reduction. Beneficial kinetics information, which is vital in the modeling of the bioremediation of these heavy metals, was also obtained from these model kinetics [164]. Therefore, a secondary modeling activity can be used to determine whether the substrate inhibits the reduction rate (Monod) or vice versa (Haldane, Teissier, Aiba, Yano, and Luong). The best model can be assessed via a comparison using statistical tests such as root mean square error (RMSE), adjusted coefficient of determination (*R^2^*), bias factor (BF), accuracy factor (AF), and corrected Akaike information criterion (AICc) [157]. It appears that the Luong model describes the effect of molybdate inhibition on Mo reduction in more bacteria than other models (Table 3). Despite the commonly reported Haldane model, the Luong and Teissier models allow for the determination of the critical concentration of substrate that can completely inhibit Mo-blue production. 

### 2.7. Characteristic of the Partially Purified Molybdenum-Reducing Enzyme from other Bacterial Sources

The first attempt to purify the Mo-reducing enzyme was first carried out in *E. cloacae strain 48* [83]. The purification began with ammonium sulfate fractionation followed by gel filtration using a Sephadex G-200, but a poor result was obtained. The partially purified enzyme was discovered to be heat-labile with a gradual loss of about half of the initial activity within 10 min at 40 °C and a total loss of activity at 100 °C within 1 min of incubation. The optimum temperature and pH for the enzymatic activity were 30 °C and 8.0, respectively. From the Lineweaver–Burk plot, the rate of molybdate (M^+6^) reduction was shown to increase with the concentration and halted as the concentration reached 100 mM. The *K_m_* (apparent) was 16.5 mM, and *V_max_* was 0.33 mU mg^−1^. A second attempt at purifying the Mo-reducing enzyme [84] from *E. cloacae* strain 48 used phosphomolybdate (PM) as an electron acceptor using ammonium sulfate (40%–50%) fractionation, and ion exchange on DEAE-cellulose and gel filtration on Sephacryl S-200. An excellent ammonium sulfate fraction was then obtained with 6.5-fold enzyme purification and about 97% recovery of the initial value. A single peak was produced from ion exchange chromatography on DEAE-cellulose. Though the level of purification was outstanding, a significant decrease in the activity was recorded in both the gel filtration and ion exchange steps. About 1.6% of the final yield was found in the initial crude, indicating that a large degree of enzyme denaturation or loss occurred, and the final specific activity was 240 mU mg^−1^ protein. A 40-fold purification was achieved after the ion exchange and gel filtration chromatographic steps. From the SDS-PAGE of the concentrated gel filtration step, three protein bands with estimated molecular weights of 95, 100, and 105 kDa were obtained, indicating that the purification of the enzyme was not successful [84].

The first purification of the molybdenum-reducing enzyme was reported from the bacterium *Serratia* sp. Dr.Y5 [137]. The enzyme activity was eluted from a Mono Q anion exchange column at 330 mM of NaCl with a gradient from 0 to 500 mM NaCl. The final step involved gel filtration on Zorbax GF-250. The molecular weight (apparent) was estimated to be 100 kDa, and it was found to be a monomeric protein. Similar to the previous results, a significant reduction in enzyme activity after ion exchange chromatography was observed [137]. 

Characterizing the purified fraction from strain Dr.Y5 showed that the optimum pH was 6 and that temperature stability occurred between 25 and 35 °C. At 15 mM 12-phosphomolybdate, a plot of initial rates against concentrations of substrate showed an apparent *V_max_* for NADH of 12 mU mg^−1^ protein and an apparent *K_m_* of 0.79 mM, whereas at 5 mM NADH, the apparent *V_max_* and apparent *K_m_* values for 12-MoP (phosphomolybdate) were 12.05 mU mg^−1^ protein and 3.87 mM, respectively. A higher *V_max_* for NADH was reported in strain Dr.Y5 than EC 48 at 6.28 mU mg^−1^ protein [137]. However, the apparent *K_m_* for NADH was lower than that of EC 48 at 1.65 mM, thus indicating a better affinity to a Mo-reducing enzyme in strain Dr.Y5. Both strains produced similar apparent *V_max_* values for 12-MoP. A higher apparent *K_m_* for 12-MoP in Dr.Y5, relative to 0.32 mM for EC 48, was obtained. which might suggest that the enzyme from EC 48 has a higher affinity for 12-MoP than strain Dr.Y5. In addition, the catalytic efficiency (*k_cat_/K_m_*) of the Mo-reducing enzyme from Dr.Y5 was found to be 5.47 M^−1^s^−1^, which is about 2 × 10^5^ less efficient than chromate reductase from *Thermus scotoductus,* a related metal reductase [172]. To date, the molybdenum-reducing enzyme from EC 48 appears to be the most efficient, with the highest *V_max_* and comparatively very low *K_m_* for NADH and phosphomolybdate [95].

The molybdenum-reducing enzyme was also successfully purified from *Serratia* sp. strain MIE [172], with steps involving an ammonium sulphate fractionation (between 40% and 50%) and gel filtration (GF-250). A final purification of 20.8-fold was obtained. The enzyme was monomeric and had a molecular weight of 100 kDa, an optimum pH of 5.0, and an optimum temperature of 35 °C for maximum activity. The *K_m_* and *V_max_* for NADH and phosphomolybdate were 0.859 mM and 16.11 mU mg^−1^ and 6.02 mM and 6.89 mU mg^−1^, respectively. The catalytic efficiency measured as the ratio of *K_cat_/K_m_* was 7.89 M^−1^s^−1^. The most recent purification of the Mo-reducing enzyme came from a cyanide-degrading bacterium *Serratia* sp. strain UPM-FR1 [123]. The purification steps involved a 50%–60% ammonium sulphate fraction and gel filtration (GF-250). A much lower final purification of 5.69-fold was obtained. Similar to other works, the enzyme was monomeric and had a molecular weight of 100 kDa, an optimum pH of 5.0, and a temperature of between 25 and 35 °C for maximum activity. The *K_m_* and *V_max_* for NADH and phosphomolybdate were 1.81 mM and 21.2 mU mg^−1^ and 4.53 mM and 21.66 mU mg^−1^, respectively, and the catalytic efficiency (*K_cat_/K_m_*) was 5.35 M^−1^s^−1^ (Table 4). The work of Opperman et al. [173] on the identification of the chromate reductase in *Thermus scotoductus* SA-01 showed that the enzyme is not novel; rather, it is an existing enzyme called the Old Yellow enzyme with a novel chromate reductase activity. This is the reason why chromate reductase does not have an enzyme commission (E.C.) number. The only established toxic metal reductases are mercury reductase [174] and selenate reductase [175]. Hence, the identification of the Mo-reducing enzyme via the sequencing of the purified enzyme is vital.

### 2.8. Use of Electron Transport Chain Inhibitors in Probing the Location of Molybdenum Reduction

The electron transport chain (ETC) is a series of electron transfer reactions taking place within the inner mitochondrial membrane. Several heme-proteins, soluble enzymes, coenzymes, Fe–S clusters, and a host of several metal cofactors are involved in the electron transfer process and subsequently form the components of the system. The components of the electron transport chain are then organized in a unique manner to form complexes (complexes I, II, III, and IV), with ATPase serving as the fifth complex (complex V) [176]. Examples of the most significant known inhibitors of respiratory enzymes include chemical agents such as amytal, rotenone, antimycin A, carbon monoxide, sodium azide, and cyanides. Rotenone inhibits NADH dehydrogenase, whereas sodium azide and cyanide are the inhibitors of cytochrome oxidase. Moreover, both antimycin A and hydroxyquinoline-N-oxide are cytochrome b inhibitors, and all of these inhibitors have been tested for their capacity to inhibit Mo reduction, with cyanide found to be the sole inhibitor of molybdenum reduction [72]. The inhibition by cyanide of Mo reduction in *E. cloacae* 48 indicates that the location occurred downstream from cytochrome [72]. However, all of the respiratory inhibitors tested on the recently isolated Mo-reducing bacteria showed no inhibition of the Mo-reducing enzyme [66,67,68,93,94,96,101,102,129]. The use of cyanide has only recently been discovered to increase the pH of the reaction mixture because it transforms the reaction mixture into a highly alkaline solution. Because phosphomolybdate is highly unstable under alkaline conditions, no reduction was observed when the effect of cyanide on *E. cloacae* strain 48 was revisited by adjusting the reaction mixture after addition of cyanide to a neutral pH [176]. Therefore, the electron transport chain is unlikely to be the location of molybdate reduction.

### 2.9. Identification of the Mo-Reducing Enzyme

The optimization of Mo-blue reduction for the purpose of bioremediation offers a method for the removal of molybdenum pollutants in addition to physical and chemical methods. Presently, molybdenum pollutants are chemically treated as a remediation technique [52]. Microbial-based molybdenum bioremediation offers several advantages over chemical-based remediation. First, Mo-blue is colloidal and forms a precipitate with bacterial biomasses [177]. This is suitable for bioremediation via entrapment with membranes, in which cells are either enclosed or immobilized. The main challenge in the elucidation of the mechanism of reduction is the identification of the enzyme. Though the enzyme has been purified and characterized in one bacterium [137], the sequencing of the enzyme was not successful.

## 3. Conclusions

This review aimed to provide an update on the current knowledge regarding the molybdenum-reducing bacteria that have been isolated to date. This review also provided an update on the ability of some molybdenum-reducers to degrade other xenobiotics, which is a feature that needs to be studied and optimized in the future. A number of these xenobiotics, such as amides, can be used as electron donors for molybdenum reduction, and the ability of other xenobiotics to act as electron donors should be studied in the future. The inhibitory effect on the molybdenum reduction of cationic heavy metals, such as mercury, copper, and silver, is also seen in other anionic reductions, such as chromate and arsenate microbial reductions. Thus, means to combat this issue need to be studied for real remediation applications in the future. The true identity of the molybdenum-reducing enzyme is currently unknown, and the future sequencing of the purified enzyme could reveal the underlying mechanism behind the reduction process. Inhibition kinetics studies have shown that molybdate is toxic at high concentrations. Thus, the limits of the capability of bacteria to remediate sites highly contaminated by molybdenum, in addition to molybdenum-rich effluent from mine tailings, should be studied. The activation energy for molybdenum reduction also needs to be explored in future studies to distinguish molybdenum from other anionic metal reductions and other xenobiotic degradation processes.

## Figures and Tables

**Table 2:** Mo-blue production models used in some previous studies.

Model	*p*	Equation	Best Model for Mo-Reducing Bacterium	Reference
Modified Logistic	3	$y=\frac{A}{\left\{1+\exp\left[\frac{4\mu_{m}}{A}(\lambda -t)+2\right]\right\}}$	nil	
Modified Gompertz	3	$y=A\exp\left\{-\exp\left[\frac{\mu_{m}^{}e}{A}(\lambda -t)+1\right]\right\}$	*Bacillus amyloliquefaciens* strain Neni-9 *Bacillus* sp. strain Neni-12 *Serratia* sp. strain HMY1 *Burkholderia* sp. strain Neni-11 *Bacillus* sp. strain Zeid 14	[79,80,119,131,134]
Modified Richards	4	$y=A{\left\{1+v\exp(1+v)\exp\left[\frac{\mu_{m}}{A}(1+v)\left(1+\frac{1}{v}\right)(\lambda -t)\right]\right\}}^{\left(\frac{-1}{v}\right)}$	nil	
Modified Schnute	4	$y=\left(\mu_{m}\frac{\left(1-\beta \right)}{\alpha }\right){\left[\frac{1-\beta \exp(\alpha \lambda +1-\beta -\alpha t)}{1-\beta }\right]}^{\frac{1}{\beta }}$	nil	
Baranyi–Roberts	4	$y=A+\mu_{m}t+\frac{1}{\mu_{m}}ln\left(e^{-\mu_{m}t}+e^{-h_{0}}-e^{-\mu_{m}t-h_{o}}\right)$ $-\ln\left(1+\frac{e^{\mu_{m}t+\frac{1}{\mu_{m}}\ln\left(e^{-\mu_{m}t}+e^{-h_{0}}-e^{-\mu_{m}t-h_{0}}\right)}-1}{e^{\left(y_{\max}-A\right)}}\right)$	nil	
Von Bertalanffy	3	$y=K{\left[1-\left[1-{\left(\frac{A}{K}\right)}^{3}\right]\exp^{-\left(\mu_{m}t/3K^{\frac{1}{3}}\right)}\right]}^{3}$	nil	
Huang	4	$y=A+y_{\max}-\ln\left(e^{A}+\left(e^{Y_{\max}}-e^{A}\right)e^{-\mu_{m}B\left(t\right)}\right)$ $B\left(t\right)=t+\frac{1}{\alpha }\ln\frac{1+e^{-\alpha \left(t-\lambda \right)}}{1+e^{\alpha \lambda }}$	*Serratia* sp. strain Dr.Y5	[139]
Buchanan Three-phase linear model	3	*y* = *A*, if *x* < *lag* *y* = *A* + *k*(*x* − *λ*), if *λ* ≤ *x* ≤ *x_max_* *y = y_max_*, if *x* ≥ *x_max_*	nil	

Note: *A* = Mo-blue lower asymptote; *q_m_* = maximum specific Mo-blue production rate; *v* = effects near which asymptote maximum Mo-blue production occurs; *P* = no of parameters; *λ* = lag time; *y_max_* = Mo-blue upper asymptote; *e* = exponent (2.718281828); *t* = sampling time; *α*, *β*, and *k* = curve fitting parameters; *h*_0_ = a dimensionless parameter quantifying the initial physiological state of the reduction process. The lag time (h^−1^) can be calculated as equal to *h*_0_/*q_max_.*

**Table 3:** Various mathematical models developed for degradation kinetics involving substrate inhibition.

Author	*p*	First Reported by	Reduction Rate	Best Model for Mo-Reducing Bacterium	Reference
Monod	2	[165]	$q_{\max}\frac{S}{K_{s}+S}$	*Enterobacter cloacae*	[111]
Haldane	3	[166]	$q_{\max}\frac{S}{S+K_{s}+\frac{S^{2}}{K_{i}}}$	Nil	
Teissier-Edward	3	[167]	$q_{\max}\left(1-\exp\left(-\frac{S}{K_{i}}\right)-\exp\left(\frac{S}{K_{s}}\right)\right)$	*Morganella* sp.	[78]
Aiba	4	[168]	$\mu_{\max}\frac{S}{K_{s}+S}\exp\left(\frac{-S}{K_{i}}\right)$	*Pantoea* sp. strain HMY-P4	[110]
Yano and Koga	4	[169]	$\frac{q_{\max}S}{S+K_{s}+\left(\frac{S^{2}}{K_{1}}\right)\left(1+\frac{S}{K}\right)}$	Nil	
Han and Levenspiel	5	[170]	$q_{\max}{\left[1-\left(\frac{S}{S_{m}}\right)\right]}^{n}\left[\frac{S}{S+K_{s}{\left(1-\frac{S}{S_{m}}\right)}^{m}}\right]$	Nil	
Luong	4	[171]	$q_{\max}\frac{S}{S+K_{s}}\left[1-{\left(\frac{S}{S_{m}}\right)}^{n}\right]$	*Bacillus pumilus* strain lbna *Bacillus* sp. strain A.rzi *Serratia* sp. strain HMY3	[68,103,123]

Note: *q_max_* = maximal reduction rate (h^−1^); *K_s_* = half saturation constant for maximal reduction (mM); *S_m_* = maximal concentration of substrate tolerated and (mM); m, n, and K = curve parameters; *S =* substrate concentration (mM); *P =* product concentration (mM).

**Table 4:** Characteristic of the partially purified molybdenum-reducing enzyme from other bacterial sources.

Bacteria	Molecular Weight	pH	Temp °C	*K_m_* NADH (mM)	*V_max_* NADH mU mg^−1^	*K_m_* PM (mM)	*V_max_* PM mU mg^−1^	*K_cat_*/*K_m_* M^−1^s^−1^	Ref.
*Enterobacter cloacae* strain 48	n.p but 80, 90, and 100 bands observed	6.5	25	1.38	102.6	2.56	99.4	n.a.	[29]
*Pseudomonas* sp. strain DRY1	n.p.	6.0	20	4.68	26.98	3.52	23.48	n.a.	[93]
*Serratia* sp. strain DRY5	105	6.0	25 to 35	0.79	12	3.87	12.05	5.47	[137]
*Bacillus pumilus* strain Lbna	n.p	5.5	25 to 35	6.646	0.057	3.399	0.106	n.a.	[135]
*Serratia sp. strain* MIE2	100	5.0	35	0.859	16.11	6.02	6.89	7.89	[172]
*Serratia* sp. strain HMY1	100	5.5	25 to 35	1.81	21.2	4.53	21.66	5.35	[123]

Note: n.p. = not purified; PM = phosphomolybdate; n.a. = not available.

## Data Availability

There were no new data generated in this study.

## References

[1] Aftab A., Memon A.R., Shah S.F.A., Memon H.R., Dahot M.U. (2013). Determination of hydrocarbons and isolation of inherent bacteria from crude oil-contaminated soil for ex-situ bioremediation. Sindh Univ. Res. J..

[2] Aftab A., Shah S.F.A., Aziz S., Memon H.U.R., Dahot M.U. (2016). Bioaugmentation of hydrocarbons using bacterial species extracted from oil contaminated site. Sindh Univ. Res. J..

[3] Ilyin I., Rozovskaya O., Travnikov M., Varygina M. (2015). Heavy Metals: Analysis of Long-Term Trends, Country-Specific Research and Progress in Mercury Regional and Global Modelling.

[4] Meyer M., Pesch R., Schröder W., Steinnes E., Uggerud H.T. (2014). Spatial patterns and temporal trends of heavy metal concentrations in moss and surface soil specimens collected in Norway between 1990 and 2010. Environ. Sci. Eur..

[5] Kaplan D., Richmond A., Hu Q. (2013). Absorption and adsorption of heavy metals by microalgae. Handbook of Microalgal Culture: Applied Phycology and Biotechnology.

[6] Soares E.V., Soares H.M.V.M. (2012). Bioremediation of industrial effluents containing heavy metals using brewing cells of *Saccharomyces cerevisiae* as a green technology: A review. Environ. Sci. Pollut. Res..

[7] Dixit R., Malaviya D., Pandiyan K., Singh U.B., Sahu A., Shukla R., Singh B.P., Rai J.P., Sharma P.K., Lade H. (2015). Bioremediation of heavy metals from soil and aquatic environment: An Overview of principles and criteria of fundamental processes. Sustainability.

[8] Rasheed A., Ghous T., Khan M., Ullah R.S. (2012). A simple flow injection pH indicator method for the determination of metal ions by urease inhibition study. J. Chem. Soc. Pak..

[9] Sulaiman M.R. (2013). Update of mercury in fish with a focus on its current status in Malaysia. J. Environ. Bioremediation Toxicol..

[10] Abdullah A., Hamzah Z., Saat A., Wood A.K., Alias M. (2015). Accumulation of mercury (Hg) and methyl mercury (MeHg) concentrations in selected marine biota from Manjung coastal area. Malays. J. Anal. Sci..

[11] Samadi N., Ansari R., Khodavirdilo B. (2018). A suitable method for removing of heavy metal ions from aqueous solutions using proper copolymer and its derivations. Eurasian J. Anal. Chem..

[12] Francisco R., Alpoim M.C., Morais P.V. (2002). Diversity of chromium-resistant and -reducing bacteria in a chromium-contaminated activated sludge. J. Appl. Microbiol..

[13] Retamal-Morales G., Mehnert M., Schwabe R., Tischler D., Zapata C., Chávez R., Schlömann M., Levicán G. (2018). Detection of arsenic-binding siderophores in arsenic-tolerating actinobacteria by a modified CAS Assay. Ecotoxicol. Environ. Saf..

[14] Hofmann M., Heine T., Malik L., Hofmann S., Joffroy K., Senges C.H.R., Bandow J.E., Tischler D. (2021). Screening for microbial metal-chelating siderophores for the removal of metal ions from solutions. Microorganisms.

[15] Sarubbo L.A., Rocha R.B., Luna J.M., Rufino R.D., Santos V.A., Banat I.M. (2015). Some aspects of heavy metals contamination remediation and role of biosurfactants. Chem. Ecol..

[16] Li P., Tao H. (2013). Cell surface engineering of microorganisms towards adsorption of heavy metals. Crit. Rev. Microbiol..

[17] Singh A., Prasad S.M. (2011). Reduction of heavy metal load in food chain: Technology assessment. Rev. Environ. Sci. Biotechnol..

[18] Zhai X.-W., Zhang Y.-L., Qi Q., Bai Y., Chen X.-L., Jin L.-J., Ma X.-G., Shu R.-Z., Yang Z.-J., Liu F.-J. (2013). Effects of molybdenum on sperm quality and testis oxidative stress. Syst. Biol. Reprod. Med..

[19] Wu S., Hu C., Tan Q., Nie Z., Sun X. (2014). Effects of molybdenum on water utilization, antioxidative defense system and osmotic-adjustment ability in winter wheat (*Triticum aestivum*) under drought stress. Plant Physiol. Biochem..

[20] Pandey R., Singh S.P. (2002). Effects of molybdenum on fertility of male rats. BioMetals.

[21] Geng C., Gao Y., Li D., Jian X., Hu Q. (2014). Contamination investigation and risk assessment of molybdenum on an industrial site in China. J. Geochem. Explor..

[22] Rahman M.A., Ahmad S.A., Salvam S., Halmi M.I.E., Yusof M.T., Shukor M.Y., Shamaan N.A., Syed M.A. (2013). Dialysis Tubing experiment showed that molybdenum reduction in *S. marcescens* strain DrY6 is mediated by enzymatic action. J. Environ. Bioremediation Toxicol..

[23] Kosaka H., Wakita K. (1978). Some geologic features of the Mamut porphyry copper deposit, Sabah, Malaysia. Econ. Geol..

[24] Nasernejad B., Kaghazchi T., Edrisi M., Sohrabi M. (1999). Bioleaching of molybdenum from low-grade copper ore. Process Biochem..

[25] Battogtokh B., Lee J.M., Woo N. (2014). Contamination of water and soil by the Erdenet copper-molybdenum mine in Mongolia. Environ. Earth Sci..

[26] Barceloux D.G., Barceloux D. (1999). Molybdenum. J. Toxicol. Clin. Toxicol..

[27] WHO (2011). WHO/SDE/WSH/03.04/11. Molybdenum in Drinking-Water: Background Document for Development of WHO Guidelines for Drinking-Water Quality.

[28] Sebenik R.F., Burkin A.R., Dorfler R.R. (2000). Molybdenum and Molybdenum Compounds. Ullmann’s Encyclopedia of Industrial Chemistry.

[29] Shukor M.Y., Rahman M.F.A., Shamaan N.A., Lee C.H., Karim M.I.A., Syed M.A. (2008). An improved enzyme assay for molybdenum-reducing activity in bacteria. Appl. Biochem. Biotechnol..

[30] AbdEl-Mongy M.A., Shukor M.S., Hussein S., Ling A.P.K., Shamaan N.A., Shukor M.Y. (2015). Isolation and Characterization of a molybdenum-reducing, phenol- and catechol-degrading *Pseudomonas putida* strain Amr-12 in soils from Egypt. Sci. Study Res. Chem. Chem. Eng. Biotechnol. Food Ind..

[31] Wuana R.A., Okieimen F.E. (2011). Heavy metals in contaminated soils: A review of sources, chemistry, risks and best available strategies for remediation. Int. Sch. Res. Not..

[32] Olaniran A.O., Balgobind A., Pillay B. (2013). Bioavailability of heavy metals in soil: Impact on microbial biodegradation of organic compounds and possible improvement strategies. Int. J. Mol. Sci..

[33] Stafford J.M., Lambert C.E., Zyskowski J.A., Engfehr C.L., Fletcher O.J., Clark S.L., Tiwary A., Gulde C.M., Sample B.E. (2016). Dietary toxicity of soluble and insoluble molybdenum to Northern Bobwhite quail (*Colinus Virginianus*). Ecotoxicology.

[34] Miller J.K., Moss B.R., Bell M.C., Sneed N.N. (1972). Comparison of ^99^Mo metabolism in young cattle and swine. J. Anim. Sci..

[35] Robinson M.F., McKenzie J.M., Tomson C.D., van Rij A.L. (1973). Metabolic balance of zinc, copper, cadmium, iron, molybdenum and selenium in young New Zealand women. Br. J. Nutr..

[36] Schroeder H.A., Frost D.V., Balass J.J. (1970). Essential metals in Man: Selenium. J. Chronic Dis..

[37] Pitt M.A. (1976). Review molybdenum toxicity: Interactions between copper, molybdenum and sulphate. Agents Actions.

[38] Jeter M.A., Davis G.K. (1954). The effect of dietary molybdenum upon growth, hemoglobin, reproduction and lactation of rats. J. Nutr..

[39] Ratnasooriya W.D., Wadsworth R.M. (1984). Effects of prazosin on fertility of male rats. Reproduction.

[40] Lyubimov A.V., Smith J.A., Rousselle S.D., Mercieca M.D., Tomaszewski J.E., Smith A.C., Levine B.S. (2004). The effects of tetrathiomolybdate (TTM, NSC-714598) and copper supplementation on fertility and early embryonic development in rats. Reprod. Toxicol..

[41] Bersényi A., Berta E., Kádár I., Glávits R., Szilágyi M., Fekete S.G. (2008). Effects of high dietary molybdenum in rabbits. Acta Vet. Hung..

[42] Yamaguchi S., Miura C., Ito A., Agusa T., Iwata H., Tanabe S., Tuyen B.C., Miura T. (2007). Effects of lead, molybdenum, rubidium, arsenic and organochlorines on spermatogenesis in fish: Monitoring at Mekong Delta Area and In Vitro Experiment. Aquat. Toxicol..

[43] Rajagopalan K.V. (1988). Molybdenum: An essential trace element in human nutrition. Annu. Rev. Nutr..

[44] Silver S., Phung L.T. (1996). Bacterial heavy metal resistance: New surprises. Annu. Rev. Microbiol..

[45] Majak W., Steinke D., Mcgillivray J., Lysyk T. (2004). Clinical signs in cattle grazing high molybdenum forage. Rangel. Ecol. Manag..

[46] Haywood S., Dincer Z., Jasani B., Loughram M.J. (2004). Molybdenum-associated pituitary endocrinopathy in sheep treated with ammonium tetrathiomolybdate. J. Comp. Pathol..

[47] Ward G.M. (1978). Molybdenum toxicity and hypocuprosis in ruminants: A Review. J. Anim. Sci..

[48] Parimoolam S., Dahalan F.A. (2013). Physicochemical Optimization of Granular Sludge in Rubber Industrial Wastewater. J. Biochem. Microbiol. Biotechnol..

[49] Zahaba M. (2015). Luminescent bacterial testing for monitoring hydrocarbon bioremediation—A Review. J. Biochem. Microbiol. Biotechnol..

[50] Sabullah M.K., Marbawi H., Faik A.A.M., Abdullah R., Sani S.A., Japanis F.G.J., Jaganathan J.N., Julius M.W., Saini N., Roland R. (2018). Bioremediation of hydrocarbon: A mini review. J. Biochem. Microbiol. Biotechnol..

[51] Elekwachi C.O., Andresen J., Hodgman T.C. (2014). Global use of bioremediation technologies for decontamination of ecosystems. J. Bioremediation Biodegrad..

[52] Davis G.K., Merian E., Anke M., Ihnat M., Stoeppler M. (1991). Molybdenum. Metals and Their Compounds in the Environment: Occurrence, Analysis and Biological Relevance.

[53] Neunhäuserer C., Berreck M., Insam H. (2001). Remediation of soils contaminated with molybdenum using soil amendments and phytoremediation. Water Air Soil Pollut..

[54] Qadir M.A., Zaidi J.H., Ahmad S.A., Gulzar A., Yaseen M., Atta S., Tufail A. (2012). Evaluation of trace elemental composition of aerosols in the atmosphere of Rawalpindi and Islamabad using radio analytical methods. Appl. Radiat. Isot..

[55] Al Kuisi M., Al-Hwaiti M., Mashal K., Abed A.M. (2015). Spatial distribution patterns of molybdenum (Mo) concentrations in potable groundwater in Northern Jordan. Environ. Monit. Assess..

[56] Jacobs J.A., Testa S.M., Jacobs J.A., Lehr J.H., Testa S.M. (2014). Acid drainage and sulfide oxidation: Introduction. Acid Mine Drainage, Rock Drainage, and Acid Sulfate Soils: Causes, Assessment, Prediction, Prevention, and Remediation.

[57] Kargar M., Khorasani N., Karami M., Rafiee G., Naseh R. (2011). Study of aluminum, copper and molybdenum pollution in groundwater sources surrounding (Miduk) Shahr-E-Babak copper complex tailings dam. World Acad. Sci. Eng. Technol..

[58] Yu C., Xu S., Gang M., Chen G., Zhou L. (2011). Molybdenum Pollution and Speciation in Nver River Sediments Impacted with Mo Mining Activities in Western Liaoning, Northeast China. Int. J. Environ. Res..

[59] Simeonov L.I., Kochubovski M.V., Simeonova B.G. (2011). Environmental Heavy Metal Pollution and Effects on Child Mental Development.

[60] LeGendre G.R., Runnells D.D. (1975). Removal of dissolved molybdenum from wastewaters by precipitates of ferric iron. Environ. Sci. Technol..

[61] Runnells D.D., Kaback D.S., Thurman E.M., Chappel W.R., Peterson K.K. (1976). Geochemistry and sampling of molybdenum in sediments, soils, and plants in Colorado. Molybdenum in the Environment.

[62] Furber D. Is Molybdenum Lurking In Your Forages? *Canadian Cattlemen* 2009. https://www.canadiancattlemen.ca/features/is-molybdenum-lurking-in-your-forages.

[63] Yong F.S. Mamut copper mine—The untold story. Proceedings of the Minerals: Underpinning Yesterday’s Needs, Today’s Development and Tomorrow’s Growth.

[64] Yakasai H.M., Rahman M.F., Yasid N.A., Ahmad S.A., Halmi M.I.E., Shukor M.Y. (2017). Elevated molybdenum concentrations in soils contaminated with spent oil lubricant. J. Environ. Microbiol. Toxicol..

[65] Lloyd J.R. (2003). Microbial reduction of metals and radionuclides. FEMS Microbiol. Rev..

[66] Shukor M.Y., Rahman M.F., Shamaan N.A., Syed M.S. (2009). Reduction of molybdate to molybdenum blue by *Enterobacter* sp. strain Dr.Y13. J. Basic Microbiol..

[67] Halmi M.I.E., Zuhainis S.W., Yusof M.T., Shaharuddin N.A., Helmi W., Shukor Y., Syed M.A., Ahmad S.A. (2013). Hexavalent molybdenum reduction to Mo-blue by a sodium-dodecyl-sulfate-degrading *Klebsiella oxytoca* strain DRY14. BioMed Res. Int..

[68] Othman A.R., Bakar N.A., Halmi M.I.E., Johari W.L.W., Ahmad S.A., Jirangon H., Syed M.A., Shukor M.Y. (2013). Kinetics of molybdenum reduction to molybdenum blue by *Bacillus* sp. strain A.Rzi. BioMed Res. Int..

[69] Levine V.E. (1925). The Reducing Properties of microorganisms with special reference to selenium compounds. J. Bacteriol..

[70] Campbell A.M., Del Campillo-Campbell A., Villaret D.B. (1985). Molybdate reduction by *Escherichia coli* K-12 and its Chl mutants. Proc. Natl. Acad. Sci. USA.

[71] Sugio T., Tsujita Y., Katagiri T., Inagaki K., Tano T. (1988). Reduction of Mo^6+^ with elemental sulfur by *Thiobacillus ferrooxidans*. J. Bacteriol..

[72] Ghani B., Takai M., Hisham N.Z., Kishimoto N., Ismail A.K.M., Tano T., Sugio T. (1993). Isolation and characterization of a Mo^6+^-reducing bacterium. Appl. Environ. Microbiol..

[73] Tucker M.D., Barton L.L., Thomson B.M. (1997). Reduction and immobilization of molybdenum by *Desulfovibrio desulfuricans*. J. Environ. Qual..

[74] AbdEl-Mongy M.A., Aqlima S.A., Shukor M.S., Hussein S., Ling A.P.K., Shukor M.Y. (2018). A PEG 4000-degrading and hexavalent molybdenum-reducing *Pseudomonas putida* strain Egypt-15. J. Natl. Sci. Found. Sri Lanka.

[75] Huang L., Tian F., Pan Y., Shan L., Shi Y., Logan B.E. (2019). Mutual benefits of acetate and mixed tungsten and molybdenum for their efficient removal in 40 L microbial electrolysis cells. Water Res..

[76] Idris D., Gafasa M.A., Ibrahim S.S., Babandi A., Shehu D., Ya’u M., Babagana K., Mashi J.A., Yakasai H.M. (2019). *Pantoea* sp. strain HMY-P4 reduced toxic hexavalent molybdenum to insoluble molybdenum blue. J. Biochem. Microbiol. Biotechnol..

[77] Kabir Z.M., Gafasa M.A., Kabara H.T., Ibrahim S.S., Babandi A., Ya’u M., Shehu D., Abubakar S.M., Babagana K., Mashi J.A. (2019). Isolation and characterization of molybdate-reducing *Enterobacter cloacae* from agricultural soil in Gwale LGA Kano State, Nigeria. J. Environ. Microbiol. Toxicol..

[78] Mohammed S., Gafasa M.A., Kabara H.T., Babandi A., Shehu D., Ya’u M., Abubakar S.M., Babagana K., Mashi J.A., Yakasai H.M. (2019). Soluble molybdenum reduction by *Morganella* sp.. locally-isolated from agricultural land in Kano. Bioremediation Sci. Technol. Res..

[79] Rusnam, Gusmanizar N. (2019). Isolation and characterization of a molybdenum-reducing and coumaphos-degrading *Bacillus* sp. strain Neni-12 in soils from West Sumatera, Indonesia. J. Environ. Microbiol. Toxicol..

[80] Rusnam, Gusmanizar N. (2020). Isolation and Characterization of a molybdenum-reducing and carbamate-degrading *Bacillus amyloliquefaciens* strain Neni-9 in soils from West Sumatera, Indonesia. Bioremediation Sci. Technol. Res..

[81] Alhassan A.Y., Babandi A., Uba G., Yakasai H.M. (2020). Isolation and characterization of molybdenum-reducing *Pseudomonas* sp. from agricultural land in Northwest-Nigeria. J. Biochem. Microbiol. Biotechnol..

[82] Saeed A.M., Sayed H.A.E., El-Shatoury E.H. (2020). Optimizing the reduction of molybdate by two novel thermophilic bacilli isolated from Sinai, Egypt. Curr. Microbiol..

[83] Ariff A.B., Rosfarizan M., Ghani B., Sugio T., Karim M.I.A. (1997). Molybdenum reductase in *Enterobacter cloacae*. World J. Microbiol. Biotecnol..

[84] Shukor M.Y., Lee C.H., Omar I., Karim M.I.A., Syed M.A., Shamaan N.A. (2003). Isolation and characterization of a molybdenum-reducing enzyme in *Enterobacter cloacae* strain 48. Pertanika J. Sci. Technol..

[85] Lee J.D. (1977). Concise Inorganic Chemistry.

[86] Shukor M.Y., Adam H., Ithnin K., Yunus I., Shamaan N.A., Syed A. (2007). Molybdate reduction to molybdenum blue in microbe proceeds via a phosphomolybdate intermediate. J. Biol. Sci..

[87] Sims R.P.A. (1961). Formation of heteropoly blue by some reduction procedures used in the micro-determination of phosphorus. Analyst.

[88] Kazansky L.P., Fedotov M.A. (1980). Phosphorus-31 and Oxygen-17 N.M.R. Evidence of trapped electrons in reduced 18-molybdodiphosphate(V), P_2_Mo_18_O_62_^8−^. J. Chem. Soc. Chem. Commun..

[89] Chae H.K., Klemperer W.G., Marquart T.A. (1993). High-nuclearity oxomolybdenum(V) complexes. Coord. Chem. Rev..

[90] Shukor M.Y., Rahman M.F., Suhaili Z., Shamaan N.A., Syed M.A. (2009). Bacterial reduction of hexavalent molybdenum to molybdenum blue. World J. Microbiol. Biotechnol..

[91] You X.-Y., Wang H., Ren G.-Y., Li J.-J., Duan X., Zheng H.-J., Jiang Z.-Q. (2015). Complete genome sequence of the molybdenum-resistant bacterium *Bacillus subtilis* strain LM 4–2. Stand. Genom. Sci..

[92] Othman A.R., Abu Zeid I.M., Rahman M.F., Ariffin F., Shukor M.Y. (2015). Isolation and characterization of a molybdenum-reducing and Orange G-decolorizing *Enterobacter* sp. strain Zeid-6 in soils from Sudan. Bioremediation Sci. Technol. Res..

[93] Ahmad S.A., Shukor M.Y., Shamaan N.A., Mac C., Syed M.A. (2013). Molybdate reduction to molybdenum blue by an Antarctic bacterium. BioMed Res. Int..

[94] Shukor M.Y., Ahmad S.A., Nadzir M.M.M., Abdullah M.P., Shamaan N.A., Syed M.A. (2010). Molybdate reduction by *Pseudomonas* sp. strain DRY2. J. Appl. Microbiol..

[95] Shukor M.Y., Habib S.H.M., Rahman M.F.A., Jirangon H., Abdullah M.P.A., Shamaan N.A., Syed M.A. (2008). Hexavalent molybdenum reduction to molybdenum blue by *S. marcescens* strain Dr. Y6. Appl. Biochem. Biotechnol..

[96] Shukor M.Y., Rahman M.F., Suhaili Z., Shamaan N.A., Syed M.A. (2010). Hexavalent molybdenum reduction to Mo-blue by *Acinetobacter calcoaceticus*. Folia Microbiol..

[97] Masdor N., Abd Shukor M.S., Khan A., Bin Halmi M.I.E., Abdullah S.R.S., Shamaan N.A., Shukor M.Y. (2015). Isolation and characterization of a molybdenum-reducing and SDS- degrading *Klebsiella oxytoca* strain Aft-7 and its bioremediation application in the environment. Biodiversitas.

[98] Khayat M.E., Rahman M.F.A., Shukor M.S., Ahmad S.A., Shamaan N.A., Shukor M.Y. (2016). Characterization of a molybdenum-reducing *Bacillus* sp. strain Khayat with the ability to grow on SDS and diesel. Rend. Fis. Acc. Lincei.

[99] Sabullah M.K., Rahman M.F., Ahmad S.A., Sulaiman M.R., Shukor M.S., Shamaan N.A., Shukor M.Y. (2016). Isolation and characterization of a molybdenum-reducing and glyphosate-degrading *Klebsiella oxytoca* strain Saw-5 in Soils from Sarawak. Agrivita.

[100] Rahman M.F.A., Shukor M.Y., Suhaili Z., Mustafa S., Shamaan N.A., Syed M.A. (2009). Reduction of Mo(VI) by the Bacterium *Serratia* Sp. Strain DRY5. J. Environ. Biol..

[101] Yunus S.M., Hamim H.M., Anas O.M., Aripin S.N., Arif S.M. (2009). Mo(VI) Reduction to molybdenum blue by *Serratia marcescens* strain Dr. Y 9. Pol. J. Microbiol..

[102] Lim H.K., Syed M.A., Shukor M.Y. (2012). Reduction of molybdate to molybdenum blue by *Klebsiella* sp. strain Hkeem. J. Basic Microbiol..

[103] Abo-Shakeer L.K.A., Ahmad S.A., Shukor M.Y., Shamaan N.A., Syed M.A. (2013). Isolation and characterization of a molybdenum-reducing *Bacillus pumilus* strain Lbna. J. Environ. Microbiol. Toxicol..

[104] Ibrahim Y., Abdel-Mongy M., Shukor M.S., Hussein S., Ling A.P.K., Shukor M.Y. (2015). Characterization of a Molybdenum-reducing bacterium with the ability to degrade phenol, isolated in soils from Egypt. Biotechnol. J. Biotechnol. Comput. Biol. Bionanotechnol..

[105] Elangovan R., Abhipsa S., Rohit B., Ligy P., Chandraraj K. (2006). Reduction of Cr(VI) by a Bacillus Sp.. Biotechnol. Lett..

[106] Rege M.A., Petersen J.N., Johnstone D.L., Turick C.E., Yonge D.R., Apel W.A. (1997). Bacterial reduction of hexavalent chromium by *Enterobacter cloacae* strain H01 grown on sucrose. Biotechnol. Lett..

[107] Hettiarachchi G.M., Pierzynski G.M., Ransom M.D. (2000). In situ stabilization of soil lead using phosphorus and manganese oxide. Environ. Sci. Technol..

[108] Deeb B.E., Altalhi A.D. (2009). Degradative plasmid and heavy metal resistance plasmid naturally coexist in phenol and cyanide assimilating bacteria. Am. J. Biochem. Biotechnol..

[109] Halmi M.I.E., Wasoh H., Sukor S., Ahmad S.A., Yusof M.T., Shukor M.Y. (2014). Bioremoval of molybdenum from aqueous solution. Int. J. Agric. Biol..

[110] Yakasai H.M., Babandi A., Uba G. (2020). Inhibition kinetics study of molybdenum reduction by *Pantoea* sp. strain HMY-P4. J. Environ. Microbiol. Toxicol..

[111] Yakasai H.M., Babandi A., Ibrahim S. (2020). Modelling the inhibition kinetics of molybdenum reduction by the molybdate-reducing *Enterobacter cloacae*. Bull. Environ. Sci. Sustain. Manag..

[112] Kesavan V., Mansur A., Suhaili Z., Salihan M.S.R., Rahman M.F.A., Shukor M.Y. (2018). Isolation and characterization of a heavy metal-reducing *Pseudomonas* sp. strain Dr.Y Kertih with the ability to assimilate phenol and diesel. Bioremediation Sci. Technol. Res..

[113] Nordmeier A., Woolford J., Celeste L., Chidambaram D. (2017). Sustainable batch production of biosynthesized nanoparticles. Mater. Lett..

[114] Saeed A.M., El Shatoury E., Hadid R. (2019). Production of molybdenum blue by two novel molybdate-reducing bacteria belonging to the genus *Raoultella* isolated from Egypt and Iraq. J. Appl. Microbiol..

[115] Gafasa M.A., Ibrahim S.S., Babandi A., Abdullahi N., Shehu D., Ya’u M., Babagana K., Mashi J.A., Yakasai H.M. (2019). Characterizing the molybdenum-reducing properties of *Pseudomonas* sp. locally isolated from agricultural soil in Kano Metropolis Nigeria. Bioremediation Sci. Technol. Res..

[116] Manogaran M., Ahmad S.A., Yasid N.A., Yakasai H.M., Shukor M.Y. (2018). Characterisation of the simultaneous molybdenum reduction and glyphosate degradation by *Burkholderia vietnamiensis* AQ5-12 and *Burkholderia* sp. AQ5-13. 3 Biotech..

[117] Karamba I.K., Yakasai H. (2018). Isolation and characterization of a molybdenum-reducing and methylene blue-decolorizing *Serratia marcescens* strain KIK-1 in soils from Nigeria. Bioremediation Sci. Technol. Res..

[118] Maarof M.Z., Shukor M.Y., Mohamad O., Karamba K.I., Halmi M.I.E., Rahman M.F.A., Yakasai H.M. (2018). Isolation and characterization of a molybdenum-reducing *Bacillus amyloliquefaciens* strain KIK-12 in soils from Nigeria with the ability to grow on SDS. J. Environ. Microbiol. Toxicol..

[119] Yakasai M.H., Ibrahim K.K., Yasid N.A., Halmi M.I.E., Rahman M.F.A., Shukor M.Y. (2016). Mathematical modelling of molybdenum reduction to Mo-blue by a cyanide-degrading bacterium. Bioremediation Sci. Technol. Res..

[120] Yakasai H.M., Yasid N.A., Shukor M.Y. (2018). Temperature coefficient and Q10 value estimation for the growth of molybdenum-reducing *Serratia* sp. strain HMY1. Bioremediation Sci. Technol. Res..

[121] Yakasai H., Karamba K., Yasid N., Halmi M.I.E., Rahman M.F., Ahmad S.A., Shukor M. (2019). Response surface-based optimization of a novel molybdenum-reducing and cyanide-degrading *Serratia* sp. strain HMY1. Desalination Water Treat..

[122] Sabullah M.K., Rahman M.F., Ahmad S.A., Sulaiman M.R., Shukor M.S., Gansau A.J., Shamaan N.A., Shukor M.Y. (2017). Isolation and characterization of a molybdenum-reducing and phenolic- and catechol-degrading *Enterobacter* sp. strain Saw-2. Biotropia Southeast Asian J. Trop. Biol..

[123] Yakasai M.H. (2017). Bioreduction of Hexavalent Molybdenum to Molybdenum Blue by Serratia sp. Strain Mie2 and Purification of the Molybdenum Reducing Enzyme. Ph.D. Thesis.

[124] Mansur R., Gusmanizar N., Roslan M.A.H., Ahmad S.A., Shukor M.Y. (2017). Isolation and characterisation of a molybdenum-reducing and metanil yellow dye-decolourising *Bacillus* sp. strain Neni-10 in soils from West Sumatera, Indonesia. Trop. Life Sci. Res..

[125] Yakasai M.H., Manogaran M. (2020). Kinetic modelling of molybdenum-blue production by *Bacillus* sp. strain Neni-10. J. Environ. Microbiol. Toxicol..

[126] Yakasai M.H., Rahman M.F.A., Rahim M.B.H.A., Khayat M.E., Shamaan N.A., Shukor M.Y. (2017). Isolation and characterization of a metal-reducing *Pseudomonas* sp. strain 135 with amide-degrading capability. Bioremediation Sci. Technol. Res..

[127] Chee H.-S., Manogaran M., Suhaili Z., Yakasai M.H., Rahman M.F.A., Shamaan N.A., Yasid N.A., Othman A.R. (2017). Isolation and characterisation of a Mo-reducing bacterium from Malaysian Soil. Bioremediation Sci. Technol. Res..

[128] Mohamad O., Yakasai H.M., Karamba K.I., Halmi M.I.E., Rahman M.F., Shukor M.Y. (2017). Reduction of molybdenum by *Pseudomonas aeruginosa* strain KIK-11 isolated from a metal-contaminated soil with ability to grow on diesel and sodium dodecyl sulphate. J. Environ. Microbiol. Toxicol..

[129] Halmi M.I.E., Abdullah S.R.S., Johari W.L.W., Ali M.S.M., Shaharuddin N.A., Khalid A., Shukor M.Y. (2016). Modelling the kinetics of hexavalent molybdenum (Mo^6+^) reduction by the *Serratia* sp. strain MIE2 in batch culture. Rend. Fis. Acc. Lincei.

[130] Aziz N.F., Halmi M.I.E., Johari W.L.W. (2017). Statistical optimization of hexavalent molybdenum reduction by *Serratia* sp. strain MIE2 using Central Composite Design (CCD). J. Biochem. Microbiol. Biotechnol..

[131] Mansur R., Gusmanizar N., Dahalan F.A., Masdor N.A., Ahmad S.A., Shukor M.S., Roslan M.A.H., Shukor M.Y. (2016). Isolation and characterization of a molybdenum-reducing and amide-degrading *Burkholderia cepacia* strain Neni-11 in soils from West Sumatera, Indonesia. IIOAB.

[132] Shukor M.S., Khan A., Masdor N., Halmi M.I.E., Abdullah S.R.S., Shukor M.Y. (2016). Isolation of a novel molybdenum-reducing and azo dye decolorizing *Enterobacter* sp. strain Aft-3 from Pakistan. Chiang Mai Univ. J. Nat. Sci..

[133] Rahman M.F., Rusnam M., Gusmanizar N., Masdor N.A., Lee C.H., Shukor M.S., Roslan M.A.H., Shukor M.Y. (2016). Molybdate-Reducing and SDS-Degrading *Enterobacter* sp. strain Neni-13. Nova Biotechnol. Chim..

[134] Mohd Adnan A.S., Abu Zeid I.M., Ahmad S.A., Halmi M.I.E., Abdullah S.R.S., Masdor M.A., Shukor M.S., Shukor M.Y. (2016). A Molybdenum-reducing *Bacillus* sp. strain Zeid 14 in soils from Sudan that could grow on amides and acetonitrile. Malays. J. Soil Sci..

[135] Abo-Shakeer L.K.A., Rahman M.F.A., Yakasai M.H., Bakar N.A., Othman A.R., Syed M.A., Abdullah N., Shukor M.Y. (2017). Kinetic studies of the partially purified molybdenum-reducing enzyme from *Bacillus pumilus* strain Lbna. Bioremediation Sci. Technol. Res..

[136] Shukor M.Y., Bakar N.A., Othman A.R., Yunus I., Shamaan N.A., Syed M.A. (2009). Development of an inhibitive enzyme assay for copper. J. Environ. Biol..

[137] Shukor M.Y., Halmi M.I.E., Rahman M.F.A., Shamaan N.A., Syed M.A. (2014). Molybdenum Reduction to Molybdenum Blue in *Serratia* sp. strain DRY5 is catalyzed by a novel molybdenum-reducing enzyme. BioMed Res. Int..

[138] Halmi M.I.E., Ahmad S.A., Yusof M.T., Shukor M.Y., Syed M.A. (2013). Entrapment of Mo-reducing bacterium increase its resistance towards heavy metals. Bull. Environ. Sci. Manag..

[139] Syed M.A., Shamaan N.A., Shukor M.Y. (2020). Mathematical modeling of the molybdenum blue production from *Serratia* sp. strain DRY5. J. Environ. Microbiol. Toxicol..

[140] Box G.E.P., Wilson K.B. (1951). On the experimental attainment of optimum conditions. J. R. Stat. Soc..

[141] Montgomery D.C., Runger G.C. (1994). Applied Statistics and Probability for Engineers.

[142] Sharma Y.C., Uma, Upadhyay S.N. (2009). Removal of a cationic dye from wastewaters by adsorption on activated carbon developed from coconut coir. Energy Fuels.

[143] Zin K.M., Halmi M.I.E., Abd Gani S.S., Zaidan U.H., Samsuri A.W., Abd Shukor M.Y. (2020). Microbial Decolorization of Triazo dye, Direct Blue 71: An Optimization Approach Using Response Surface Methodology (RSM) and Artificial Neural Network (ANN). BioMed Res. Int..

[144] Zwietering M.H., Jongenburger I., Rombouts F.M., Van’t Riet K. (1990). Modeling of the bacterial growth curve. Appl. Environ. Microbiol..

[145] Baranyi J., Roberts T.A. (1994). A Dynamic approach to predicting bacterial growth in food. Int. J. Food Microbiol..

[146] Buchanan R.L., Whiting R.C., Damert W.C. (1997). When is simple good enough: A comparison of the gompertz, baranyi, and three-phase linear models for fitting bacterial growth curves. Food Microbiol..

[147] Fujikawa H. (2010). Development of a new logistic model for microbial growth in foods. Biocontrol Sci..

[148] Johnsen A.R., Binning P.J., Aamand J., Badawi N., Rosenbom A.E. (2013). The Gompertz Function can coherently describe microbial mineralization of growth-sustaining pesticides. Environ. Sci. Technol..

[149] Shukor M.S., Shukor M.Y. (2015). Bioremoval of toxic molybdenum using dialysis tubing. Chem. Eng. Res. Bull..

[150] Gusmanizar N., Halmi M., Rusnam M., Rahman M., Shukor M., Azmi N., Shukor M.Y. (2016). Isolation and characterization of a molybdenum-reducing and azo-dye decolorizing *Serratia marcescens* strain Neni-1 from Indonesian soil. J. Urban Environ. Eng..

[151] Halmi M.I.E., Ahmad S.A., Syed M.A., Shamaan N.A., Shukor M.Y. (2014). Mathematical modelling of the molybdenum reduction kinetics in *Bacillus pumilus* strain Lbna. Bull. Environ. Sci. Manag..

[152] Ricker F.J., Hoar W.S., Randall D.J., Brett J.R. (1979). 11 Growth rates and models. Bioenergetics and Growth.

[153] Gompertz B. (1825). On the nature of the function expressive of the law of human mortality, and on a new mode of determining the value of life contingencies. Philos. Trans. R. Soc..

[154] Richards F.J. (1959). A flexible growth function for empirical use. J. Exp. Bot..

[155] Baranyi J. (1995). Mathematics of predictive food microbiology. Int. J. Food Microbiol..

[156] Babák L., Šupinová P., Burdychová R. (2012). Growth models of thermus aquaticus and *Thermus scotoductus*. Acta Univ. Agric. Silvic. Mendel. Brun..

[157] López S., Prieto M., Dijkstra J., Dhanoa M.S., France J. (2004). Statistical evaluation of mathematical models for microbial growth. Int. J. Food Microbiol..

[158] Buchanan R.L. (1993). Predictive food microbiology. Trends Food Sci. and Technol..

[159] Huang L. (2013). Optimization of a new mathematical model for bacterial growth. Food Control.

[160] Glusczak L., dos Santos Miron D., Crestani M., Braga da Fonseca M., de Araújo Pedron F., Duarte M.F., Vieira V.L.P. (2006). Effect of glyphosate herbicide on acetylcholinesterase activity and metabolic and hematological parameters in piava (*Leporinus obtusidens*). Ecotoxicol. Environ. Saf..

[161] Soda S.O., Yamamura S., Zhou H., Ike M., Fujita M. (2006). Reduction Kinetics of As (V) to As (III) by a Dissimilatory arsenate-reducing bacterium, *Bacillus* sp. SF-1. Biotechnol. Bioeng..

[162] Sukumar M. (2010). Reduction of hexavalent chromium by Rhizopus oryzae. Afr. J. Environ. Sci. Technol..

[163] Truex M.J., Peyton B.M., Valentine N.B., Gorby Y.A. (1997). Kinetics of U(VI) reduction by a dissimilatory Fe(III)-reducing bacterium under non-growth conditions. Biotechnol. Bioeng..

[164] King R.B., Long G.M., Sheldon J.K. (1992). Practical Environmental Bioremediation: The Field Guide.

[165] Monod J. (1949). The growth of bacterial cultures. Annu. Rev. Microbiol..

[166] Boon B., Laudelout H. (1962). Kinetics of nitrite oxidation by *Nitrobacter winogradskyi*. Biochem. J..

[167] Teissier G. (1942). Growth of bacterial populations and the available substrate concentration. Rev. Sci. Instrum..

[168] Aiba S., Shoda M., Nagatani M. (1968). Kinetics of product inhibition in alcohol fermentation. Biotechnol. Bioeng..

[169] Yano T., Koga S. (1969). Dynamic behavior of the chemostat subject to substrate inhibition. Biotechnol. Bioeng..

[170] Han K., Levenspiel O. (1988). Extended Monod kinetics for substrate, product, and cell inhibition. Biotechnol. Bioeng..

[171] Luong J.H.T. (1987). Generalization of Monod kinetics for analysis of growth data with substrate inhibition. Biotechnol. Bioeng..

[172] Halmi M.I.E. (2012). Bioreduction of Hexavalent Molybdenum to Molybdenum Blue by *Serratia* sp. Strain MIE2 and Purification of the Molybdenum Reducing Enzyme. Ph.D. Thesis.

[173] Opperman D.J., Piater L.A., van Heerden E. (2008). A novel chromate reductase from *Thermus scotoductus* SA-01 related to old yellow enzyme. J. Bacteriol..

[174] Freedman Z., Zhu C., Barkay T. (2012). Mercury resistance and mercuric reductase activities and expression among chemotrophic thermophilic aquificae. Appl. Environ. Microbiol..

[175] Schröder I., Rech S., Krafft T., Macy J.M. (1997). Purification and characterization of the selenate reductase from *Thauera selenatis*. J. Biol. Chem..

[176] Shukor M.Y. (2014). Revisiting the role of the electron transport chain in molybdate reduction by *Enterobacter cloacae* Strain 48. Indian J. Biotechnol..

[177] Shukor M.Y., Syed M.A., Lee C.H., Karim M.I.A., Shamaan N.A. (2002). A method to distinguish between chemical and enzymatic reduction of molybdenum in *Enterobacter cloacae* Strain 48. Malays. J. Biochem..

